# Understanding the Solution Behavior of Epinephrine in the Presence of Toxic Cations: A Thermodynamic Investigation in Different Experimental Conditions

**DOI:** 10.3390/molecules25030511

**Published:** 2020-01-24

**Authors:** Francesco Crea, Concetta De Stefano, Anna Irto, Gabriele Lando, Stefano Materazzi, Demetrio Milea, Alberto Pettignano, Silvio Sammartano

**Affiliations:** 1 Dipartimento di Scienze Chimiche, Biologiche, Farmaceutiche ed Ambientali, Università degli Studi di Messina, V.le F. Stagno d’Alcontres, 31, I-98166 Messina, Italy; cdestefano@unime.it (C.D.S.); airto@unime.it (A.I.); glando@unime.it (G.L.); dmilea@unime.it (D.M.); ssammartano@unime.it (S.S.); 2 Dipartimento di Chimica, Università “La Sapienza” di Roma, Piazzale A. Moro 5, I-00185 Rome, Italy; stefano.materazzi@uniroma1.it; 3 Dipartimento di Fisica e Chimica, Università degli Studi di Palermo, V.le delle Scienze, ed. 17, I-90128 Palermo, Italy; alberto.pettignano@unipa.it

**Keywords:** epinephrine, toxic cations, enthalpy and entropy changes, dependence on ionic strength, sequestering ability

## Abstract

The interactions of epinephrine ((*R*)-(−)-3,4-dihydroxy-α-(methylaminomethyl)benzyl alcohol; *Eph*^−^) with different toxic cations (methylmercury(II): CH_3_Hg^+^; dimethyltin(IV): (CH_3_)_2_Sn^2+^; dioxouranium(VI): UO_2_^2+^) were studied in NaCl_aq_ at different ionic strengths and at *T* = 298.15 K (*T* = 310.15 K for (CH_3_)_2_Sn^2+^). The enthalpy changes for the protonation of epinephrine and its complex formation with UO_2_^2+^ were also determined using isoperibolic titration calorimetry: Δ*H*_HL_ = −39 ± 1 kJ mol^−1^, Δ*H*_H2L_ = −67 ± 1 kJ mol^−1^ (overall reaction), Δ*H*_ML_ = −26 ± 4 kJ mol^−1^, and Δ*H*_M2L2(OH)2_ = 39 ± 2 kJ mol^−1^. The results were that UO_2_^2+^ complexation by *Eph*^−^ was an entropy-driven process. The dependence on the ionic strength of protonation and the complex formation constants was modeled using the extended Debye–Hückel, specific ion interaction theory (SIT), and Pitzer approaches. The sequestering ability of adrenaline toward the investigated cations was evaluated using the calculation of pL_0.5_ parameters. The sequestering ability trend resulted in the following: UO_2_^2+^ >> (CH_3_)_2_Sn^2+^ > CH_3_Hg^+^. For example, at *I* = 0.15 mol dm^−3^ and pH = 7.4 (pH = 9.5 for CH_3_Hg^+^), pL_0.5_ = 7.68, 5.64, and 2.40 for UO_2_^2+^, (CH_3_)_2_Sn^2+^, and CH_3_Hg^+^, respectively. Here, the pH is with respect to ionic strength in terms of sequestration.

## 1. Introduction

Catecholamines, such as dopamine, epinephrine (or adrenaline), and norepinephrine, are hormones produced by the medullary portion of the adrenal glands, which are small triangular organs located above the kidneys. They are released in blood in response to physical or emotional stress and help to transmit nerve impulses to the brain, increase the availability of glucose and fatty acids to promote energy production, and dilate the bronchioles and pupils. After performing their activities, catecholamines are metabolized into inactive forms: dopamine is transformed in homovanilic acid (HVA), norepinephrine turns into normetanephrine and vanilmandelic acid (VMA), and epinephrine becomes metanephrine and VMA. Both hormones and their metabolites are excreted by urine, in which they are usually present in small variable concentrations, which appreciably increase during and immediately after exposure to stress [1,2].

Pheochromocytomas and other types of neuroendocrine tumors, however, can produce large amounts of catecholamines, leading to their increased concentration levels in both blood and urine (together with their metabolites). This may result in persistent and severe episodic hypertension, severe headache, tachycardia, excessive sweating, nausea, anxiety, and tingling in the extremities.

**Scheme 1:**
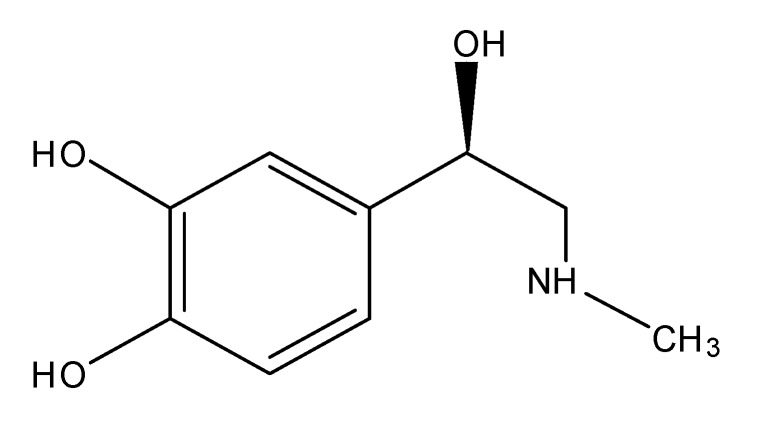
(R)-(−)-3,4-Dihydroxy-α-(methylaminomethyl)benzyl alcohol.

Epinephrine ((*R*)-(−)-3,4-dihydroxy-α-(methylaminomethyl)benzyl alcohol, also known as adrenaline, Scheme 1), is synthesized by an enzymatic process that converts tyrosine into a series of intermediates and ultimately epinephrine. In particular, epinephrine acts on nearly all body tissues and increases heart rate and the force of heart contractions, facilitating blood flow to the muscles and the brain. Epinephrine relaxes smooth muscle and helps the conversion of glycogen into glucose in the liver, increasing the blood sugar level: it also causes acceleration in breathing and in medicine is used in treatments for cardiac arrest, asthma, and glaucoma [3,4,5].

The behavior of epinephrine in physiological conditions is dependent on the different forms in which it can be present, i.e., on its chemical speciation. As an example, epinephrine can have an influence on platelet aggregation, for which epinephrine is an agonist [6], but this phenomenon is influenced by the interaction between divalent and trivalent metal ions such as calcium, copper, manganese, magnesium, cadmium, and aluminum.

As it has been reported in many papers, the behavior of catecholamines in aqueous solution depends on many parameters, such as pH, temperature, ionic medium, ionic strength, and exposure to light and oxygen (they tend to be quickly oxidized) [1,7,8,9,10,11,12].

The acid–base properties and solubility of epinephrine in different experimental conditions have already been documented in the literature [13,14,15,16,17]. Taking into account that the pharmacological action of epinephrine can be influenced by the presence of metal cations present in the same environment, we decided to investigate the complexation of this hormone toward some selected toxic cations by using (Ion Selective Electrode) ISE-[H^+^] potentiometry, UV spectrophotometry, calorimetry, and thermogravimetry at ionic strength values ranging from 0 to 1.0 *I*/mol dm^−3^ at *T* = 298.15 K and *T* = 310.15 K. Due to their importance from both a biological and environmental point of view, the selected cations were (CH_3_)_2_Sn^2+^, UO_2_^2+^, and CH_3_Hg^+^, whose properties and activities are already well known [18,19,20,21,22,23,24,25,26,27,28,29,30,31,32,33,34].

In this investigation, the speciation models of the cation–epinephrine systems and the corresponding formation constants of the species in different experimental conditions were determined following well-established criteria for the selection of the best speciation models, which have already been reported in previous papers [35,36,37,38,39].

The dependence of the formation constants on ionic strength was studied by means of a Debye–Hückel type equation, as well as through Pitzer and specific ion interaction theory (SIT) approaches [40,41,42,43,44,45,46,47,48].

The enthalpy and entropy change values of protonation for epinephrine and complex formation for the metal–ligand species of UO_2_^2+^ were also determined using isoperibol calorimetry.

Using a simple Boltzmann-type equation, the sequestering ability of epinephrine toward the investigated toxic cations was quantified in different experimental conditions by means of the pL_0.5_ parameter.

The information obtained from the speciation studies can be useful in modeling the behavior of such molecules in quite different experimental conditions, such as those of many natural waters and biological fluids.

## 2. Results and Discussion

### 2.1. Acid–Base Properties of Epinephrine and Investigated Cations

The acid–base properties of the cations investigated in this work (i.e., their hydrolytic behavior), as well as those of epinephrine (i.e., its protonation), must be known in the exact experimental conditions of the considered systems. The hydrolysis constants of (CH_3_)_2_Sn^2+^, UO_2_^2+^, and CH_3_Hg^+^ in NaCl_(aq)_ at 0 < *I*/mol dm^−3^ ≤ 1.0 and *T* = 298.15 K (*T* = 310.15 K for (CH_3_)_2_Sn^2+^) were taken from References [49,50,51] and are reported in Tables S1–S3. In the case of methylmercury(II), its chloride interactions were explicitly taken into account, considering the formation of CH_3_HgCl species through their corresponding stability constants in calculations (Table S3).

Concerning the protonation constants of epinephrine, it is worth mentioning here that, as with other catecholamines, its acid–base properties are not easy to determine, since they tend to be oxidized in aqueous solution, especially at pH > 10.0–10.5 [1,9,10,52]. Moreover, epinephrine has four potentially functional groups, that can undergo acid-base reactions, but only two are in the pH range of biological interest. For this reason, it has often been considered to be a bidentate ligand, though some studies have reported some values related to the third protonation step (log *K*_a_ ~ 13) [13,53,54] associated with the protonation/dissociation of the second phenolic group. Log *K*_a_ values of ~ 13.5–14 have been attributed to the hydroxyl group of the alkyl chain [13,53,54]. The values used in this work have been previously determined by this group together with their dependence on ionic strength and temperature in different models. Protonation constants at infinite dilution and the corresponding parameters for their dependence on ionic strength in the EDH and SIT models (Equations (4)–(7)) for values in molar and molal concentration scales, respectively, in NaCl_(aq)_ at *T* = 298.15 and 310.15 K are reported in Table S4 [17].

### 2.2. Epinephrine–Cation Interactions

Chemical speciation studies of cation/ligand systems, such as those investigated in this work, are particularly difficult, not only for the accurate determination of the stability constants of formed complexes, but also for their identification, i.e., for the definition of reliable speciation models. In this vein, some general rules and guidelines may be very helpful [35,36,37,38,39], such as the following:

(i) The simplicity of the proposed model. The simpler the better, both in terms of number and nature of species considered;
(ii) The formation percentages of the species considered in the investigated conditions. Sometimes minor species are included in models to improve the quality of fits, but their significance has to be verified, possibly in a broad range of experimental conditions and/or using different techniques;
(iii) The likelihood of the proposed species. *Similar* ligands generally form *similar* species and show *similar* binding modes. Theoretical and/or experimental evidence about coordination may be supportive; and
(iv) “Statistical significance” of species and models. Experimental data are, nowadays, almost exclusively elaborated by software based on nonlinear least squares minimization. A derived fit’s parameters (e.g., variances/standard deviations of fits, uncertainties associated with refined variables), which are obtained for various models, should be compared (by relative tests) to check for statistically significant differences.

Evidently, all this cannot prescind from a consistent number of experiments opportunely designed to cover a wide range of conditions (in terms of total concentrations, concentration ratios, and pH, as well as ionic media, temperatures, and ionic strengths, if pertinent).

#### 2.2.1. CH_3_Hg^+^/*Eph*^−^ Complexes

Investigations of a CH_3_Hg^+^/*Eph*^−^ system were carried out up to pH = 11 without observing the formation of sparingly soluble species. The stability constants of the CH_3_Hg^+^/*Eph*^−^ species, determined in NaCl_(aq)_ at different ionic strengths and at *T* = 298.15 K, are reported in Table 1.

**Figure 1:**
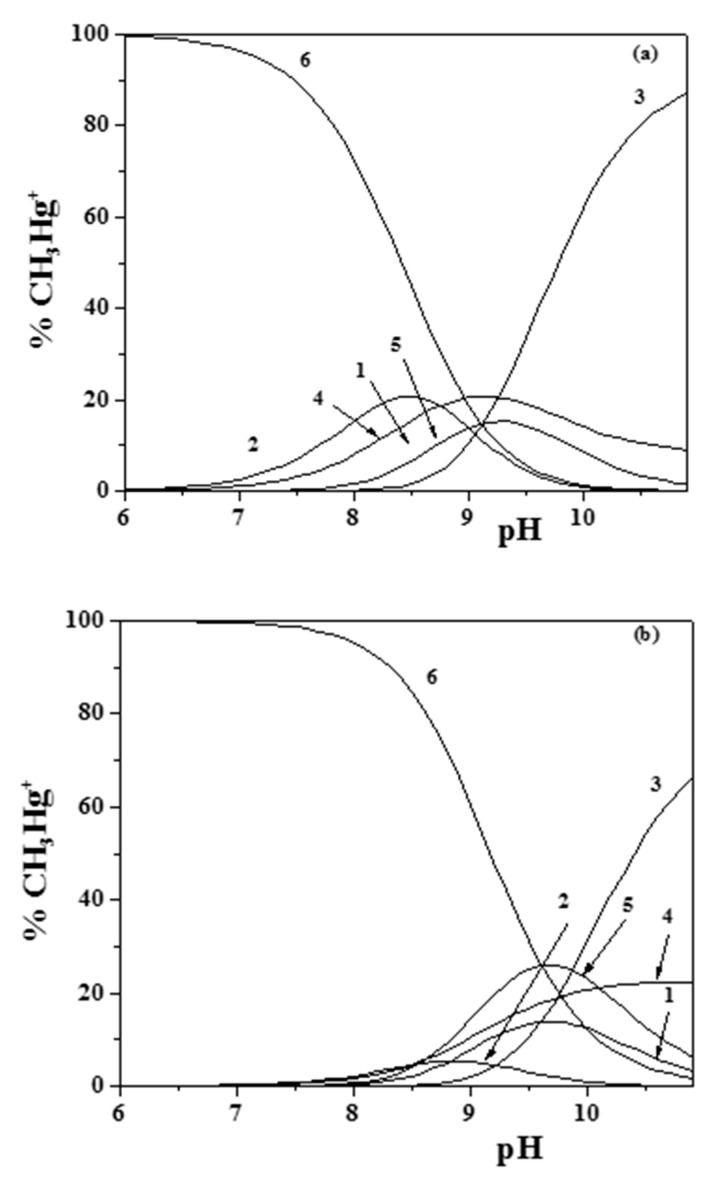
(**a**,**b**) Distribution diagram of the species for the CH_3_Hg^+^/*Eph*^−^ system: C_CH3Hg+_ = 1 mmol dm^−3^; C_Eph_ = 3 mmol dm^−3^ ((**a**) *I* = 0.15 mol dm^−3^; (**b**) *I* = 1 mol dm^−3^ (Stoichiometry of the species: 1: ML; 2: MLH; 3: MLOH; 4: MOH; 5: MLCl; 6: MCl); M = CH_3_Hg^+^; L = *Eph*^−^).

The speciation of methylmercury(II) in sodium chloride aqueous solutions was characterized by the formation of MOH and MCl species (Table S3). The latter species was quite stable, so that, in the presence of epinephrine, the formation of ternary MLCl^−^ was observed in the pH range 8.0–10.5, as is shown in the speciation diagrams in Figure 1a,b.

Concerning the other species determined (i.e., MLH^+^, ML^0^, and MLOH^−^), ML^0^ was formed almost in the same pH range as the MLCl^−^ species, while MLH^+^ occurred at lower pH values (about 7), and MLOH^−^ occurred above pH ~8.5. Comparing the diagrams in Figure 1a,b, obtained at *I* = 0.15 and *I* = 1.0 mol dm^−3^, respectively, the effect of the ionic strength on the distribution of the species can be observed, which resulted in a shift of the maximum formation percentages toward higher pH values at higher ionic strengths.

From the analysis of the distribution diagrams in Figure 1a,b, it is also evident how the formation of CH_3_Hg^+^/*Eph*^−^ species became significant at pH ~9.0–9.5. This is an indication that, in physiological conditions (e.g., in blood plasma at pH ~7.4), the behavior of adrenaline is scarcely influenced by the presence of CH_3_Hg^+^.

#### 2.2.2. (CH_3_)_2_Sn^2+^/*Eph*^−^ Complexes

The interactions between dimethyltin(IV) and epinephrine were studied at *T* = 310.15 K and in NaCl_(aq)_ at different ionic strengths by means of potentiometry, though some checks were also made at *I* = 0.15 mol dm^−3^ using UV-VIS spectrophotometric titrations (Table S5).

In the experimental conditions adopted, the formation of the MLH^2+^, ML^+^, and MLOH^0^ species was observed. The corresponding stability constants are reported in Table 2.

As it was already said, UV-VIS spectrophotometric titrations at *I* = 0.15 mol dm^−3^ and *T* = 310.15 K were also carried out, confirming the speciation model obtained using potentiometric data and obtaining stability constants that were highly comparable to the potentiometric results: log *β*_110_ = 15.74 ± 0.04, log *β*_111_ = 20.11 ± 0.03, and log *β*_11-1_ = 8.19 ± 0.02. 

**Figure 2:**
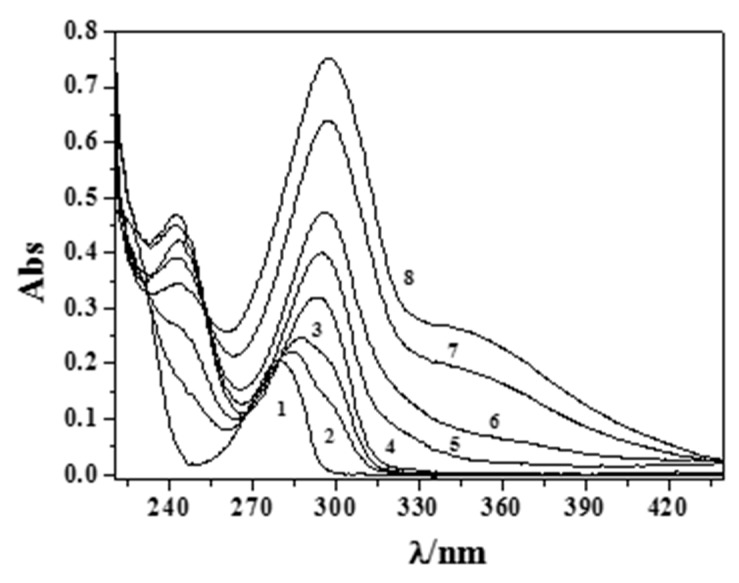
Titration curves at some different pH values at *I* = 0.15 mol dm^−3^ and *T* = 310.15 K for the (CH_3_)_2_Sn^2+^/*Eph^−^* system. Experimental conditions: c_M_ = 0.03 mmol dm^−3^ and c_L_ = 0.09 mmol dm^−3^ (1: pH = 2.38; 2: pH = 7.37; 3: pH = 8.14; 4: pH = 9.14; 5: pH = 10.06; 6: pH = 10.36; 7: pH = 10.92; 8: pH = 11.21).

An example of spectrophotometric titration is reported in Figure 2 (experimental conditions: *c*_M_ = 0.03 mmol dm^−3^; c*_Eph_* = 0.09 mmol dm^−3^), in which spectra were collected from pH = 2.38 to pH = 11.21 without the formation of sparingly soluble species being observed.

**Figure 3:**
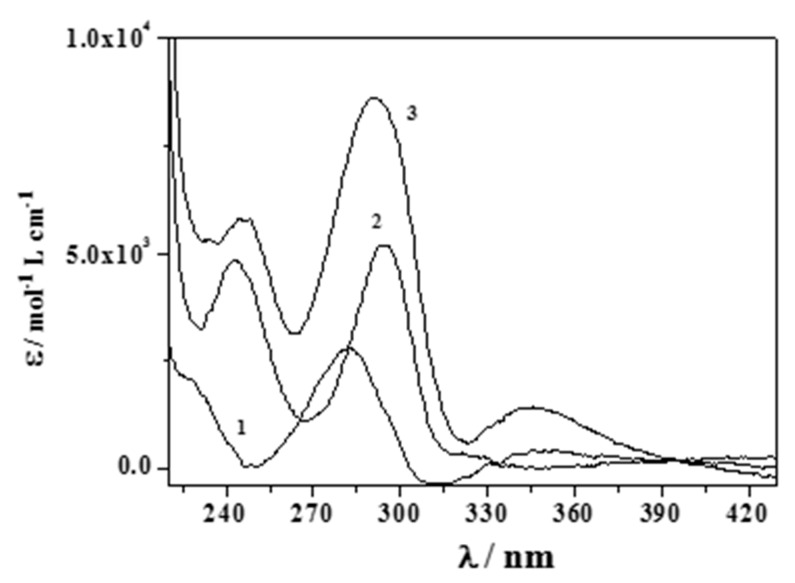
Here, ε/mol^−1^ L cm^−1^ versus λ/nm for the (CH_3_)_2_Sn^2+^/*Eph^−^* species in NaCl_aq_ at *I* = 0.15 mol dm^−3^ and *T* = 310.15 K (Stoichiometry of the species. 1: MLH; 2: ML; 3: MLOH; M = (CH_3_)_2_Sn^2+^; L = *Eph^−^*).

Increasing pH and a bathochromic shift of the band at λ_max_ = 279 nm were observed up to λ ~295 nm, together with a hypsochromic shift for the band at λ = 248 nm. The molar extinction coefficients (*ε*/mol^−1^ cm^−1^ dm^−3^) of the free and protonated adrenaline species were already determined in a prior paper [17] (Figure S1), while those relative to the (CH_3_)_2_Sn^2+^ complexes are studied here and are reported in Figure 3. Comparing the determined data using the different analytical techniques and taking into account the different concentration range used, we can consider them to be in very good agreement.

**Figure 4:**
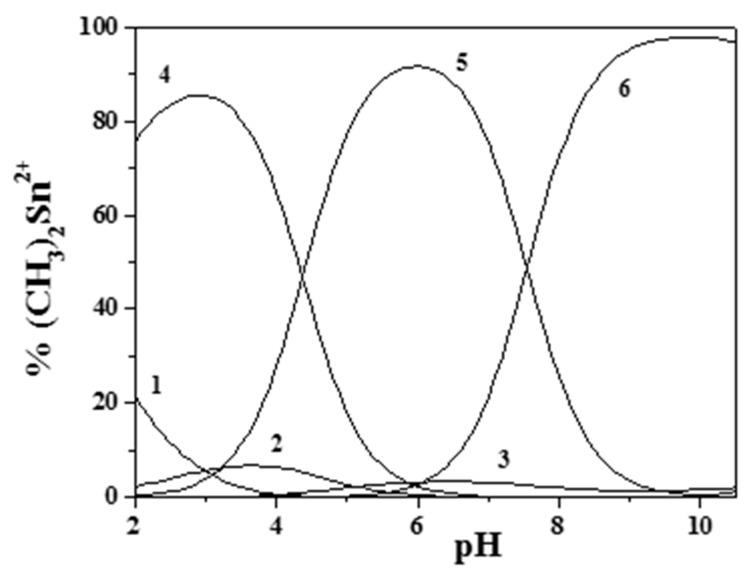
Distribution diagram of the species for the (CH_3_)_2_Sn^2+^/*Eph^−^* system at *I* = 0.15 mol dm^−3^, *T* = 310.15 K, and c_M_ = c_L_ = 3.00 mmol dm^−3^ (from potentiometric data). (Stoichiometry of the species. 1: M; 2: M(OH); 3: M(OH)_2_; 4: MLH; 5: ML; and 6: MLOH. M = (CH_3_)_2_Sn^2+^; L = *Eph^−^*).

**Figure 5:**
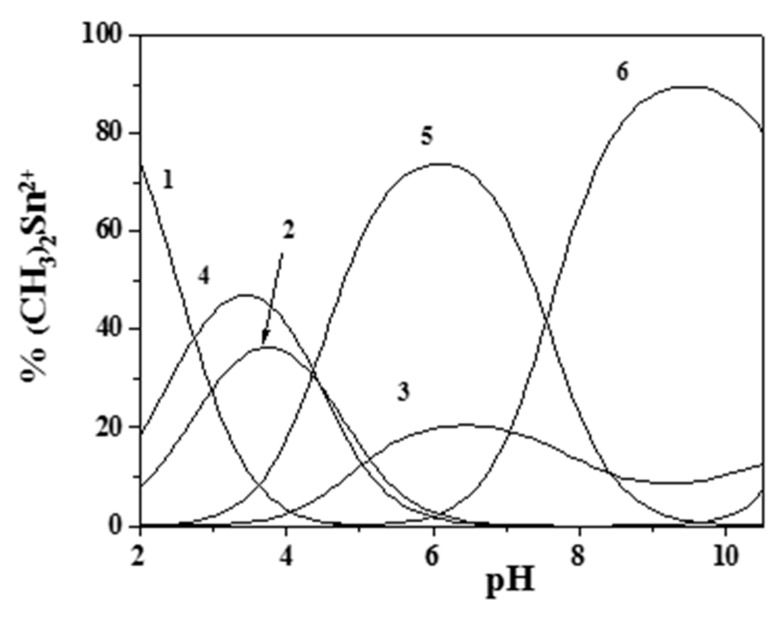
Distribution diagram of the species for the (CH_3_)_2_Sn^2+^/*Eph^−^* system at *I* = 0.15 mol dm^−3^, *T* = 310.15 K, and c_M_ = c_L_ = 0.06 mmol dm^−3^ (from UV spectrophotometric data). (Stoichiometry of the species. 1: M; 2: M(OH); 3: M(OH)_2_; 4: MLH; 5: ML; 6: MLOH. M = (CH_3_)_2_Sn^2+^; L = *Eph^−^*).

Figure 4 and Figure 5 report the distribution diagrams of the (CH_3_)_2_Sn^2+^/*Eph^−^* species in NaCl_(aq)_ at *I* = 0.15 mol dm^−3^ and *T* = 310.15 K in a 1:1 molar ratio at two different concentrations. 

As can be observed, a higher presence of both the free metal ion (at low pH) and the hydrolytic species of (CH_3_)_2_Sn^2+^ occurred at low component concentrations (UV-VIS spectrophotometric measurements). Nevertheless dimethyltin(IV)/epinephrine species were significant in all of the investigated pH range, an indication that this cation may influence epinephrine behavior in physiological conditions.

#### 2.2.3. UO_2_^2+^/*Eph*^−^ Complexes

As reported in the experimental section, dioxouranium(VI) was used in the form of UO_2_(*Ac*)_2_ salt, meaning that acetate was always present during the experiments at a concentration always double that of UO_2_^2+^. Since this cation tends to form quite stable complexes with many carboxylic ligands, including acetate, the stability constants of UO_2_^2+^/*Ac*^−^ complexes must be taken into account during data analysis. Corresponding values were taken from a previous work [55] and are reported in Table S6. An analysis of experimental data for the UO_2_^2+^/*Eph*^−^ system at *T* = 298.15 K in NaCl_(aq)_ at different ionic strengths was performed up to pH ~8.5, since the formation of sparingly soluble species was observed in all conditions at higher pH values. The resulting elaboration was particularly difficult due to the tendency of UO_2_^2+^ to form further species with stoichiometries different from mononuclear/monomeric ones. By applying the above-cited criteria for the selection of the most suitable speciation model, the formation of many different species was considered and tested. Finally, the accepted speciation scheme accounted for the formation of four species, namely the mononuclear ML^+^ and MLOH and the dinuclear M_2_L_2_^2+^ and M_2_L_2_(OH)_2_. The corresponding stability constants at different ionic strengths are reported in Table 3.

**Figure 6:**
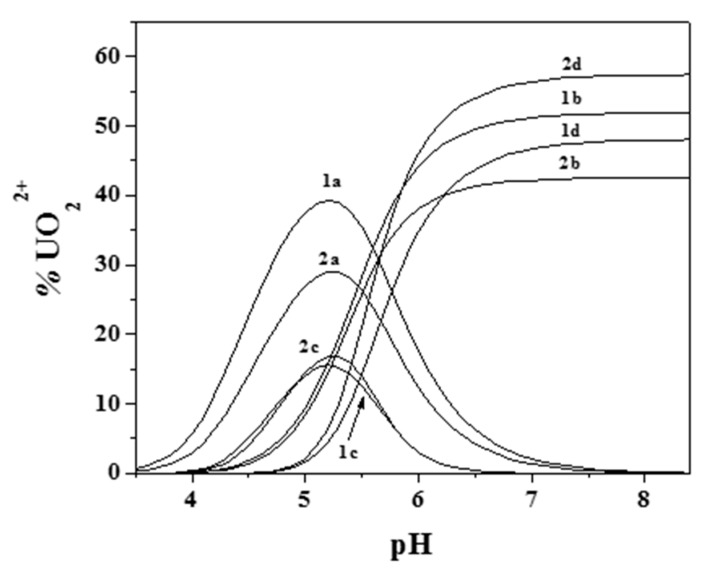
Distribution diagram of the species for the UO_2_^2+^/*Eph*^−^ system: c_UO22+_ = 1 mmol dm^−3^ e; c_Eph_ = 3 mmol dm^−3^ (1: *I* = 0.15 mol dm^−3^; 2: *I* = 1 mol dm^−3^; (Stoichiometry of the species. a: ML; b: MLOH; c: M_2_L_2_; d: M_2_L_2_(OH)_2_. M = UO_2_^2+^; L = *Eph^−^*).

**Figure 7:**
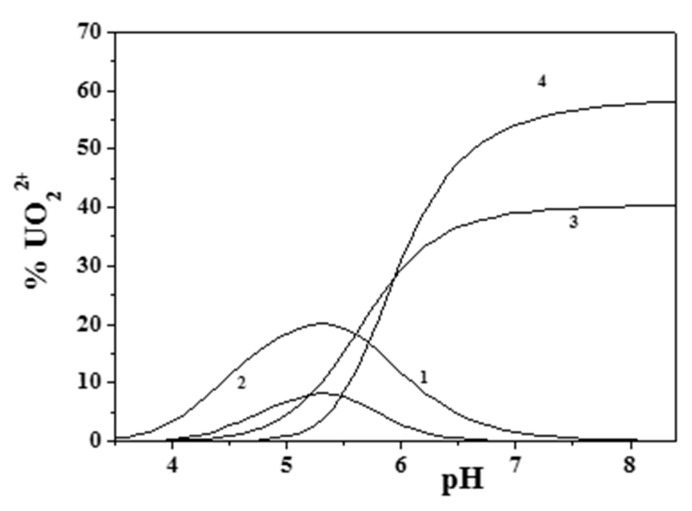
Distribution diagram of the species for the UO_2_^2+^/*Eph*^−^ system at *I* = 0.15 mol dm^−3^. c_UO22+_ = 2 mmol dm^−3^ and c_Eph_ = 2 mmol dm^−3^ (Stoichiometry of the species. 1: ML; 2: M_2_L_2_; 3: MLOH; 4: M_2_L_2_(OH)_2_. M = UO_2_^2+^; L = *Eph^−^*).

Figure 6 and Figure 7 better evidence the effect of ionic strength and of the ligand-to-metal ratio on the distribution of the species for the UO_2_^2+^/*Eph*^−^ system. As was observed, dioxouranium(VI) complexation by epinephrine became significant at pH > 4.0, with all corresponding species reaching high formation percentages. At pH ~7.4, UO_2_^2+^ speciation was dominated by the formation of the M_2_L_2_(OH)_2_ species, which reached percentages higher than 40%.

Changes in ionic strength (Figure 6) only affected the formation percentages of the species and had little/no effect on the pH range in which they were present, which also happened when varying the ligand-to-metal ratio (Figure 7).

### 2.3. Calorimetric Analysis

By means of an isoperibol titration calorimeter, the enthalpy changes for the protonation epinephrine and its complex formation with UO_2_^2+^ were determined in NaCl_(aq)_ at *I* = 0.5 mol dm^−3^ and *T* = 298.15 K by the heat of reactions collected during the titrations.

Concerning the determination of the protonation enthalpy changes of epinephrine, solutions containing *Eph^−^* (4–5 mmol dm^−3^) and the ionic medium were previously neutralized with standard NaOH solution and then titrated with standard HCl solutions, as is described in the experimental section. The ligand concentrations reported above were used in order to obtain suitable amounts of the LH^0^ and LH^2+^ species during the calorimetric titrations, allowing for the measurement of the Δ*H*/kJ mol^−1^ (expressed as overall formation enthalpies according to L^−^ + i H^+^ = LH_i_^(i−1)^, where L = generic ligand; H = proton). The protonation enthalpy changes of epinephrine are reported in Table 4 and were in good agreement with analogous values reported in the literature for similar molecules, such as dopamine (Δ*H* = −46 and −83 kJ mol^−1^ for LH^0^ and LH_2_^+^, respectively) [53,56].

The protonation enthalpy changes determined, as described above, were then used as input in calculations for the determination of formation enthalpy changes of UO_2_^2+^/*Eph*^−^ species, together with those related to UO_2_^2+^ hydrolysis and its acetate complexes (taken from the literature) [55,57]. The formation thermodynamic parameters are reported in Table 4 only for the ML and M_2_L_2_(OH)_2_ species, since no reliable data could be obtained for other complex species in the experimental conditions adopted. The reliability of the results obtained is also supported by the low values of both the standard deviation for the global fit of experimental data (*σ* = 0.054) and the mean deviation of the variation of the heats of reaction (∂Q = 0.146). As can be observed, the formation of the ML species was exothermic, while the overall formation reaction of M_2_L_2_(OH)_2_ was endothermic. Even considering the reaction relative to the hydrolysis and dimerization of the ML complex as it formed M_2_L_2_(OH)_2_ species (according to the reaction 2 ML = M_2_L_2_(OH)_2_ + 2 H^+^), it was Δ*H* = 91 kJ mol^−1^. These values, when analyzed with all of the thermodynamic parameters reported in Table 4, clearly indicate how the main contribution to complex formation is entropic in nature.

### 2.4. Ionic Strength Dependence

The dependence of formation constants on ionic strength was investigated by means of an extended Debye–Hückel (EDH)-type equation, SIT (specific ion interaction theory), and the Pitzer approach. Further details are given in their dedicated section.

The stability constants of cation/epinephrine complexes in Table 1, Table 2 and Table 3 were fitted to Equation (4) to obtain their corresponding values at infinite dilution and their *C* parameters (EDH) to model their dependent ionic strength on a molar scale, as reported in Table 5.

The same table also reports the analogous Δ*ε* values (SIT) obtained from the fitting of constants after their conversion to the molal concentration scale [58,59,60]. These parameters could be used for the calculation of the complex formation constants of M^n+^/*Eph*^−^ species at ionic strengths different from those experimentally investigated (from infinite dilution up to *I* ~ 1.0) on the molar and/or molal concentration scales. Some calculated values are reported in Tables S7–S9.

As reported in the experimental section, Δ*ε* values account for all the classical SIT coefficients (*ε*) of species involved in the considered equilibrium. The calculation of Δ*ε* instead of classical SIT coefficients is particularly indicated when one or more *ε* values are not available (so that the system of equations related to various equilibria results is undetermined, hampering the calculation of other single *ε* values), as it is preferable to resorting to assumptions/approximations that could lead to erroneous results (as could happen, for example, when the activity coefficients of neutral species are fixed as γ = 1). Nevertheless, when it is possible, the calculation of classical SIT coefficients is desired, as they are of more general utility. In this vein, the SIT coefficients of single UO_2_^2+^/*Eph*^−^ species were calculated in this work (Table 6), while this is not possible for CH_3_Hg^+^/*Eph*^−^ and (CH_3_)_2_Sn^2+^/*Eph*^−^ systems.

Analogous considerations can be done when adopting the Pitzer approach. Instead of making unsuitable approximations and/or assumptions, the simplified Pitzer equation (Equation (20)) can be used instead of classical Pitzer formalism (Equations (10)–(18)). The simplified Pitzer coefficients for the dependence of the stability constants of all the epinephrine/cation species (including protonation constants) determined in this work are reported in Table 7.

As noted, fits were performed, refining only *p*_1_ and *p*_3_ parameters and neglecting *p*_2_. The rationale behind this choice was that *p*_2_, dependent on *I*^2^, accounts for the *C*^Φ^ and Ψ coefficients in classical Pitzer equations, which can usually be neglected at relatively low ionic strengths (usually at *I* < 1 mol kg^−1^). Moreover, it is known that classical Pitzer coefficients are very highly correlated, so that their simultaneous refinement dramatically increases the risk of overparametrization [64]. That is why, when possible, and when no statistically significant improvements of fit quality can be obtained, it is preferable to reduce the number of Pitzer coefficients that are calculated, as was done in this work.

However, in addition to the simplified Pitzer approach, classical Pitzer coefficients were determined for the protonation constants of epinephrine (with a standard deviation on the whole fit of *σ* = 0.083) and for the UO_2_^2+^/*Eph*^−^ species (*σ* = 0.054), analogously to what was done for the SIT model. These values are reported in Table 8.

### 2.5. Sequestering Ability

For accurate comparisons between different metal/ligand systems in terms of sequestration, comparing the stability of some common species to the same stoichiometry is not always sufficient, because secondary interactions of both the metal and the ligand under consideration with other components in the systems can influence the effective strength of the interaction between them.

Other difficulties are observed when the sequestering ability has to be estimated at different pH values, ionic strengths, and temperatures. To facilitate this kind of evaluation, the use of the parameter pL_0.5_ has been proposed. This represents the total concentration of ligands necessary to sequester 50% of a metal cation present in trace concentration (~10^−10^ mol dm^−3^) in a given solution. This parameter is described through a sigmoidal-type Boltzmann equation:

(1) $$x_{M}=\;\frac{1}{{1+10}^{{(pL-pL}_{0.5})}},$$

where *x*_M_ is the mole fraction of metal complexed by the ligand, pL = -log *c*_L_, and pL_0.5_ = -log *c*_L_ when *x*_M_ = 0.5. The sequestering ability can be graphically represented by a dose–response curve characterized by asymptotes equal to 1 for pL→−∞ and 0 for pL → +∞, which are obtained by plotting the mole fraction of the metal complexed versus the pL values. The higher the value of pL_0.5_, the greater the sequestering ability is. With this method of calculation (further details can be found in Reference [33]), pL_0.5_ allows for an evaluation of the sequestering ability of a ligand in any condition, such as at different pHs, ionic strengths, ionic media and temperatures and/or in the presence of any interfering ligands and/or metal cations, as often occurs in real multicomponent systems. Moreover, it is independent of the analytical concentration of the metal ion (since it is considered to be a trace concentration).

The pL_0.5_ values for the sequestration of epinephrine by the metal ions were investigated and calculated in various conditions, and they are reported in Table 9.

**Figure 8:**
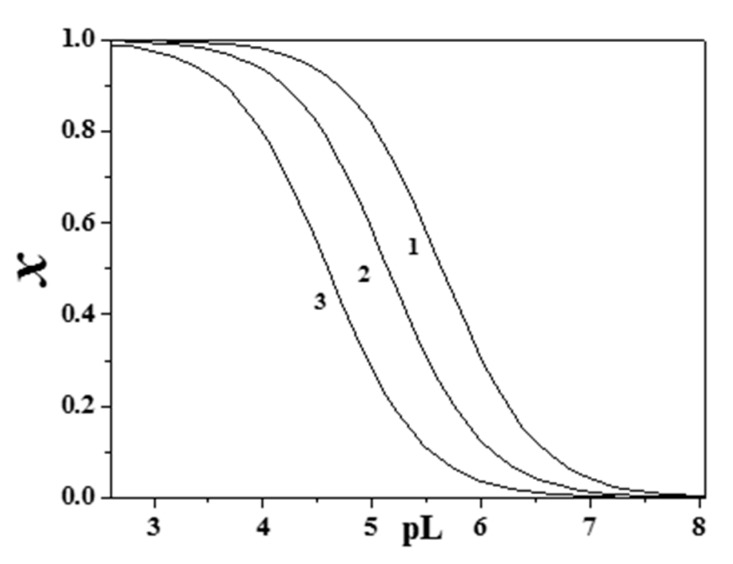
Sequestering ability of *Eph^−^* toward (CH_3_)_2_Sn^2+^ at *T* = 310.15 K, pH = 7.4 (1: *I* = 0.15 mol dm^−3^, pL_0.5_ = 5.64; 2: *I* = 0.50 mol dm^−3^, pL_0.5_ = 5.15; 3: *I* = 1.00 mol dm^−3^, pL_0.5_ = 4.59).

The sequestering ability of epinephrine toward CH_3_Hg^+^ became significant above pH ~9.0–9.5, since at lower pH values this cation is almost entirely complexed by chloride ions (see Figure 1a,b), especially at high ionic strength. At the same pH value, the sequestering ability of *Eph*^−^ toward UO_2_^2+^ was fairly constant, with a mean value of pL_0.5_ = 7.80 ± 0.09 at pH = 7.4. For (CH_3_)_2_Sn^2+^ at *T* = 310.15 K, a variation of about one order of magnitude with ionic strength was observed at pH = 7.4 (see Figure 8).

**Figure 9:**
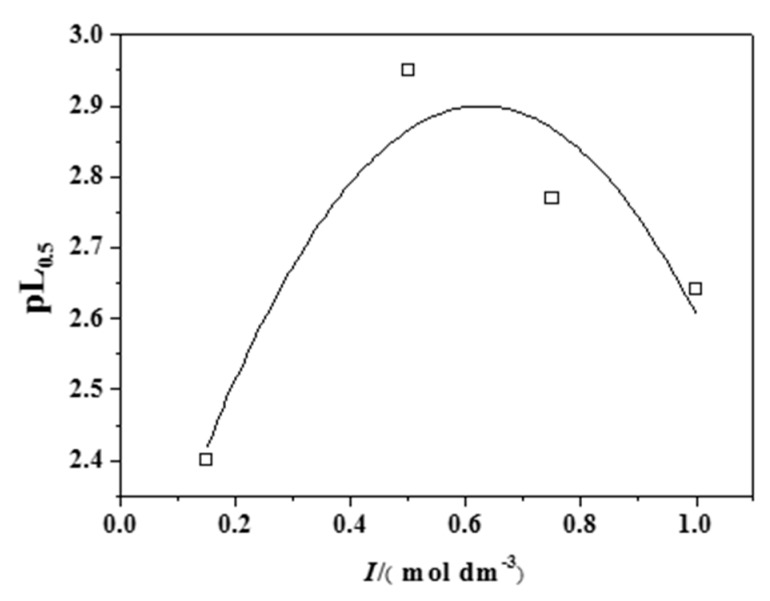
Trend of the pL_0.5_ values for the CH_3_Hg^+^/*Eph*^−^ system at different ionic strengths and *T* = 298.15 K.

In the case of CH_3_Hg^+^, an initial increase of the pL_0.5_ with the ionic strength was observed up to about *I* = 0.5 mol dm^−3^, while it started to decrease above this value (see Figure 9). This was justified by the formation of a CH_3_HgCl^0^ species, which lowered the free cation concentration and consequently the extent of its interaction with epinephrine.

However, the effect of pH on pL_0.5_ was more significant than that of ionic strength (see Figures S2 and S3 for (CH_3_)_2_Sn^2+^ and UO_2_^2+^, respectively), with the sequestering ability generally increasing with pH due to the ligand deprotonation, though the greater cation hydrolysis competed with sequestration (epinephrine increased pH). In any case, in the analysis of pL_0.5_ values calculated in various conditions, the following trend could be generally observed concerning the sequestering ability of *Eph*^−^ toward the investigated cations: UO_2_^2+^ >> (CH_3_)_2_Sn^2+^ >> CH_3_Hg^+^.

### 2.6. Thermogravimetric Characterization

A series of UO_2_^2+^/*Eph*^−^-precipitated compounds was investigated by thermogravimetric analysis (TGA) to determine stoichiometry and thermal stability. This information can be useful in comparing the solution and solid state properties of characterized complexes.

**Figure 10:**
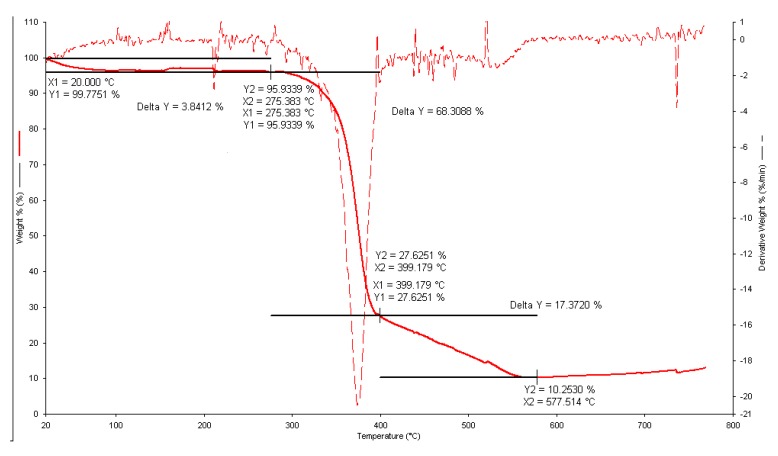
Thermogravimetric curve for the UO_2_^2+^/*Eph*^−^ precipitate obtained at *I* = 0.15 mol dm^−3^. Component concentration in the solution: C_UO22+_ = 2 mmol dm^−3^ and C_Eph_ = 4 mmol dm^−3^. Solid line: % weight loss versus *T*/°C; dashed line: derivative weight loss versus *T*/°C.

The TGA profiles in an oxidant purging atmosphere (air flow) always showed a similar stability of the precipitated solids up to 275 °C, followed by two well-defined weight loss steps (see, as an example, Figure 10). The first one occurred in the temperature range 275–400 °C, and the second, final process occurred in the temperature range 400–580 °C. The percent weight loss from the TGA curves was calculated and related to the molecular weight of the ligand to propose the stoichiometry of the analyzed precipitates. These results allowed for a determination of the number of ligand molecules lost in the initial TGA step and the final decomposition (to give uranyl oxide).

The UO_2_^2+^/*Eph*^−^ ratios in the precipitated solids were very different and were systematically dependent on the solution conditions (i.e., ionic strength). However, the calculated stoichiometry of the analyzed precipitates highlighted an unusual coordination that is realistically not possible, since the experimental evidence showed that the dependence of the stoichiometric uranyl/ligand ratios on the ionic strength of the starting solution led to an UO_2_^2+^/*Eph*^−^ ratio up to 1:13. The calculations performed on the precipitate obtained at *I* = 0.15 mol dm^−3^ (pH of formation ~8.5) allowed us to calculate a metal-to-ligand molar ratio of 3:10, assuming the formation of U_3_O_8_ at the end of the thermal decomposition. An elaboration performed on the precipitate, obtained at *I* = 0.75 mol dm^−3^, led to a metal-to-ligand molar ratio of 1:7.

The experimental evidence led to the conclusion that all of these precipitates were the result of a coprecipitation process that was the consequence of the stability of aggregates in solution due to increasing solution interactions, which were favored by the increasing ionic strength. The experimental evidence also elicited that the formation and precipitation of solid state structures was relatively fast, and the precipitated solids were consequently influenced.

### 2.7. Literature Comparisons

To our knowledge, no thermodynamic studies on the interaction of epinephrine with the cations under investigation have ever been reported in literature. The only information for the UO_2_^2+^/*Eph*^−^ system has been with regard to spectral studies. Some papers have reported results on epinephrine interactions with different ions, but only at a single ionic strength and temperature. Some of them are shown in Table 10.

The main difficulty in a direct comparison to the results obtained here was due to the different approaches used for the determination of the acid–base properties of epinephrine. In some cases, it has been considered to be a triprotic ligand (L^2-^) using the calculation of the third protonation constant (with a log *K*^H^ value of about 13–14), which is out of the pH range of physiological interest [13,53,54,56]. In other cases [17], only the protonation constants of one phenolic group and of the amine group of the alkyl chain have been considered. Other aspects that must be taken into account during comparisons are the different ionic media and ionic strengths used and the pH range of investigation, which can influence the obtained results. The literature has essentially been with regard to the interaction of epinephrine with divalent metal cations (Mn^2+^, Co^2+^, Ni^2+^, Zn^2+^, Cu^2+^, Cd^2+^, Pb^2+^, Hg^2+^), even if some data are also available for lanthanide complexes.

In a comparison between the speciation models and the stability constants reported in Table 10, a fairly good agreement in terms of simplicity of the speciation schemes (characterized by simple mononuclear species) can be observed. The behavior of the UO_2_^2+^/*Eph*^−^ system has been different in terms of formation of binuclear species with stoichiometry: M_2_L_2_ and M_2_L_2_(OH)_2_, as is usual for the chemistry of UO_2_^2+^. Similar considerations can be done comparing the stability constants of the most common MLH species.

Again, no data have been published for both the dependence of the stability constants on ionic strength and for the determination of the complex formation enthalpy changes. The only available data that are useful for comparison are with regard to the protonation enthalpy changes of dopamine [53,56]. Considering the similarity of its structure to epinephrine, it is possible to state that the results here obtained can be considered to be in good agreement.

Taking into account the order of magnitude of the stability of complexes formed by epinephrine with the cations here investigated and comparing it to information from the literature, it is possible to hypothesize that for our systems as well, the interaction should occur via the phenolic oxygen(s) of the two phenolic groups, excluding the amine group of the lateral chain [13,65,66,67,69,70,71]. There has been various evidence that has supported this assumption, as has been reported by Moustafa [71], who carried out FT-IR spectra on an Hg^2+^/*Eph*^−^ complex. He observed that, in this complex, characteristic bands of epinephrine at 3331 cm^−1^ and 1340 cm^−1^, which were ascribable to the stretching and bending of the catechol groups, disappeared in the spectrum of the binary metal complex due to the displacement of the hydrogens by the metal ion and consequent coordination through the oxygen of the phenolic groups. Moustafa also showed that in the FT-IR spectra of the binary Hg^2+^/*Eph*^−^ complex, the presence of a characteristic band of adrenaline at 3448 cm^−1^ was due to an –OH of the side chain ethanolamine. This is a further indication that only two phenolic groups are involved in coordinating with metal ions. Besides, Jameson and Neille [65] have stated that Ni^2+^/*Eph*^−^ complexes have anomalous behavior, explaining that there is chelation through the interaction of the phenolic and secondary amine groups, even if it occurs only at given metal-to-ligand molar ratios.

## 3. Materials and Methods

### 3.1. Chemicals

Epinephrine solutions were prepared by weighing the ligand without further purification. The purity was checked potentiometrically by alkalimetric titrations and was >99%. Sodium chloride aqueous solutions were prepared by weighing pure salt previously dried in an oven at *T* = 383.15 K for 2 h. Sodium hydroxide and hydrochloric acid solutions were prepared from concentrated ampoules and standardized against potassium hydrogen phthalate and sodium carbonate, respectively. Solutions of (CH_3_)_2_Sn^2+^ and CH_3_Hg^+^ were prepared from the corresponding chlorides or nitrate salts and were used without further purification. For UO_2_^2+^, diacetate salt was used, and the purity was determined through the gravimetric determination of uranium after ignition to the oxide U_3_O_8_ [55]_._ All products were purchased from Sigma-Aldrich (Milan, Italy) (only dimethyltin(IV) was from Alfa-Aesar (Gandle, Germany)) and its various brands at their highest available purity. All solutions were prepared with analytical-grade water (*ρ* = 18 MΩ cm^−1^) using grade A glassware and were preserved from atmospheric CO_2_ by means of soda lime traps.

### 3.2. Apparatus and Procedure

#### 3.2.1. Potentiometric Titrations

The interactions of epinephrine with the selected cations were studied potentiometrically by means of an apparatus consisting of an 809 model Metrohm Titrando system connected to a half-cell Ross Type glass electrode (model 8101 from Thermo-Orion (Waltham, MA, USA)) coupled with a standard Ag/AgCl reference electrode. The system, which was connected to a personal computer and controlled by a Metrohm TiAMO 2.2 computer program, allowed us to carry out automatic titrations through the addition of the desired amounts of titrant when the equilibrium state was reached and to record the e.m.f. (electromotive force) of the solution under investigation. The estimated accuracy was ±0.15 mV and ±0.003 mL for the e.m.f and titrant volume readings, respectively. The measurements were carried out under magnetic stirring in thermostat cells at *T* = 298.15 and 310.15 ± 0.1 K by means of water circulation in the outer chamber of the titration cell (from a thermocryostat (model D1-G Haake)). Purified N_2(g)_ was bubbled into the solutions in order to exclude the presence of CO_2(g)_ and O_2(g)_. The titrant solutions consisted of different amounts of the desired cation, epinephrine, an excess of hydrochloric acid, and NaCl to obtain the desired ionic strength values. In order to investigate the possible formation of both mono- and polynuclear species, solutions were prepared in a wide range of cation-to-ligand molar ratios and were titrated with standard, carbonate-free NaOH up to alkaline pH values or until the formation of sparingly soluble species. As an example, Table 1 reports the experimental conditions employed in the investigations of the (CH_3_)_2_Sn^2+^/*Eph*^−^ system (component concentrations, ligand/cation molar ratios, titrant concentrations, pH ranges of investigation, mean number of experimental points collected for each titration, and number of measurements for each experimental condition). They are for *I* = 0.15 mol dm^−3^, but similar conditions were also used at the other investigated ionic strengths, as well as for the CH_3_Hg^+^/ and UO_2_^2+^/*Eph*^−^ systems. For each experiment, independent titrations of strong acid (HCl) solutions with NaOH solutions were carried out under the same experimental conditions of the metal/ligand system, with the aim of determining the electrode potential (*E*^0^) and the acidic junction potential (*E*_j_ = *j*_a_ [H^+^]). In this way, the pH scale used was a free concentration scale, pH ≡ −log [H^+^], where [H^+^] is the free proton concentration (not activity). The reliability of the calibration in the alkaline range was checked by calculating the ionic product of water (p*K*_w_).

#### 3.2.2. Spectrophotometric Titrations

The spectrophotometric measurements, carried out at *T* = 310.15 ± 0.1 K, were performed by a Varian Cary 50 (Agilent Scientific Instruments (Santa Clara, CA, USA)) UV-VIS spectrophotometer equipped with an optic fiber probe with a fixed 1-cm path length. A wavelength range from λ = 200 to 450 nm was investigated. The spectrophotometer was connected to a Personal Computer, and Varian Cary WinUV (3.00 version) software was used for the data acquisition (absorbance (*A*) versus wavelength (λ/nm)). During these measurements, a 602 Biotrode combined metro-sensor glass electrode (from Metrohm (Herisau, Switzerland)) was inserted into the thermostat measurement cell (total volume of 25 or 50 mL). The electrode was connected to a 713-model Metrohm potentiometer, and the addition of titrant was carried out by a 665-model Metrohm automatic burette. This allowed us to record simultaneously for each addition of titrant: absorbance (*A*) versus wavelength (λ/nm) (from the spectrophotometric apparatus) and e.m.f. (mV) versus the volume of titrant (mL) (from the potentiometric apparatus). The solutions under investigation consisted of different amounts of dimethyltin(IV), epinephrine, and background salt to reach pre-established ionic strength values (see Table S5 for experimental conditions). The homogeneity of the solution during the titration was performed with a magnetic stirring bar, and before each experiment, N_2(g)_ was bubbled in the solution for at least 5 min in order to exclude the presence of CO_2(g)_ and O_2(g)_.

#### 3.2.3. Calorimetric Titrations

Calorimetric titrations were performed at *T* = 298.150 ± 0.001 K by a Calorimetry Sciences Corporation (CSC, Lindon, UT, USA) Model 4285 calorimeter equipped with a Mod. 7211 constant temperature bath, both for the determination of the protonation enthalpy changes of epinephrine and the corresponding values for complexation with UO_2_^2+^. In the first case, 25 or 50 mL of solution containing epinephrine in variable concentrations (from 4 to 5 mmol dm^−3^), previously neutralized with NaOH and NaCl_(aq)_ in order to obtain a pre-established ionic strength value (*I* = 0.5 mol dm^−3^), were titrated with HCl (*c*_H_ = 0.5133 mol dm^−3^), which was delivered by a 2.5-cm^3^-capacity Hamilton syringe, model 1002TLL (Sigma Aldrich, Milan, Italy). The pH range investigated was from pH ~10.5 up to pH ~4.8 (see Table S10 for experimental conditions). For each experimental condition, measurements were repeated at least three times. For the enthalpy change values of the complexation of epinephrine with UO_2_^2+^, a different procedure was used. The titrand solutions consisted of epinephrine in variable concentrations (from 4 to 6 mmol dm^−3^) previously neutralized with NaOH (for protonation measurements and NaCl_(aq)_) in order to have a pre-established ionic strength value (*I* = 0.5 mol dm^−3^). The titrant was the salt of the metal ion, in this case UO_2_(Ac)_2_ (dioxouranium diacetate salt). The enthalpy of dilution was measured before each experiment. The accuracy of the calorimetric apparatus was *Q* ± 0.008 J, and the accuracy of the titrant volume was ±0.001 cm^3^. This was checked by titrating a THAM (tris-(hydroxymethyl)amino-methane) buffer with HCl. The enthalpy changes used in the calculations for the ionization of water at different ionic strengths were taken from De Stefano et al. [72].

#### 3.2.4. Thermogravimetric Measurements

A thermoanalytic characterization was performed using Perkin-Elmer TGA7 (Waltham, MA, USA) equipment. The investigated samples (approximately 2–10 mg) were heated in platinum crucibles in the temperature range 20–850 °C under an atmosphere of air (gaseous mixture of nitrogen and oxygen with 80% and 20% v/v, respectively) at a flow rate of 100 mL min^−1^ and a scanning rate of 10 °C min^−1^. These conditions allowed for the best resolution of the thermogravimetric curves.

### 3.3. Calculations

#### 3.3.1. Computer Programs

BSTAC and STACO computer programs were used for the refinement of all the parameters (formation constants, analytical concentration of reagents, formal electrode potential) of the alkalimetric titrations. The least squares computer program LIANA, which refines the parameters of a generic *y* = f(*x*) linear or nonlinear equation, was used to fit the Debye–Hückel, Pitzer, and SIT parameters and to calculate the formation constants at infinite dilution through an extrapolation of the experimental ones at different ionic strengths. Details for the BSTAC, STACO, and LIANA computer programs are reported in Reference [73]. UV spectra were analyzed by the Hypspec2014 computer program [74], which allows for the calculation of stability constants and the molar absorbance spectrum of each absorbing species using the experimental absorbance intensity, the analytical concentration of reagents, and the proposed chemical model as input. The advantage of this program is that for aqueous solutions containing few components, it allows for the simultaneous treatment of potentiometric and spectrophotometric data. The ES4ECI program [75] was used for the calculation of the formation percentages of the species present in solution at equilibrium and to draw both the speciation and the sequestration diagrams in different conditions. The ES5CM computer program [76] was used for the elaboration of calorimetric data from the isoperibol titration calorimetry. The input data for the elaboration of the calorimetric data contained the hydrolysis constants of uranyl and their corresponding enthalpy change values, the protonated species of epinephrine, the UO_2_ acetate species, and the formation constants of the UO_2_^2+^/*Eph^−^* complexes (determined by potentiometry). This software allows for the determination of enthalpy changes in solution from calorimetric data. The main characteristics of the program for the calculation of the Δ*H* for the equilibria involved in solution were (i) the calculation of the concentrations of all the species at each point of the calorimetric titration and (ii) the resolution of the linear equations system:

(2) $$-Q_{corr,h}=\sum_{i}\Delta H_{i}^{0}\delta n_{ih},$$

where *Q_corr_* is the heat of the reaction collected and corrected for the dilution and for the contribution to the heat of the reaction due to the species for which the Δ*H^0^* is known; *δ_n_* is the concentration variation for the *i*th species; and *h* is the index for the point of titration.

Within the manuscript, if not differently specified, hydrolysis (*q* = 0, *r* < 0) constants of cations, protonation (*p* = 0) constants of the ligands (*L^z^*^−^), and complex formation constants are given according to the overall equilibrium:

p M^m+^ + q L^z−^ + r H^+^ = M_p_L_q_H_r_^(mp−zq+r)^   log *β*_pqr._ (3)

Ligand protonation constants may also be given according to the stepwise equilibrium:

H^+^ + LH_r−1_^(z−r−1)−^ = LH_r_^(z−r)−^   log *K*_01r._ (4)

Protonation, hydrolysis and complex formation constants, concentrations, and ionic strengths are expressed in the molar (*c*, mol dm^−3^) or molal (*m*, mol kg^−1^(H_2_O)) concentration scales. Molar to molal conversions were performed using appropriate procedures that have already been reported in previous papers [58,59,60]. In the manuscript, if not differently reported, the errors associated with formation constants, enthalpy and entropy change values, and the parameters for dependence on ionic strength are expressed as a 95% confidence interval (C.I.).

#### 3.3.2. Dependence on Ionic Strength

The dependence of the formation constants on ionic strength was studied by means of different approaches, namely the extended Debye–Hückel (EDH), specific ion interaction theory (SIT), and Pitzer approaches (general information can be found, e.g., in References [40,41,42,43,44,45,46], while for some examples of their applications one can refer to [64,77,78,79,80]).

##### Extended Debye–Hückel (EDH) and Specific Ion Interaction Theory (SIT) Approaches

The extended Debye–Hückel-type equation used was

(5) $$\log\;\beta_{pqr}^{}=\log\beta_{pqr}^{0}-A\;z^{*}\;\frac{\sqrt{I}}{1+1.5\;\sqrt{I}}+C\;I,$$

where $\beta_{pqr}^{}$ and $\beta_{pqr}^{0}$ refer to the formation constant of the M*_p_*L*_q_*H*_r_* species at a given ionic strength and at infinite dilution, respectively;

(6) $$A\;=\;0.51\;+\;\frac{0.856\cdot \left(T-298.15\right)\;+\;0.00385\cdot {\left(T-298.15\right)}^{2}}{1000};$$

(7) $$z^{*}=\sum^{\;}z_{react}^{2}-\sum^{\;}z_{prod}^{2};$$

and *C* is an empirical parameter that accounts for the dependence of the formation constants on ionic strength in the molar concentration scale [81]. When stability constants and ionic strengths are expressed in the molal concentration scale, Equation (4) becomes the classical and widely used SIT (specific ion interaction theory) equation, in which *C* is replaced by Δ*ε*, where

(8) $$\Delta \varepsilon =\sum \varepsilon_{react}-\sum \varepsilon_{prod}$$

and *ε* is the SIT coefficient for the interaction of all ionic species involved in the considered equilibrium with all the ions (of opposite sign) of the ionic medium. In all equilibria involving uncharged species, their activity coefficients must also be taken into account by the Setschenow equation [62,82],

(9) $$\log\gamma =k_{c,m}I,$$

in which *k*_c_ and *k*_m_ are the Setschenow coefficients of the neutral species in a given medium in the molar and molal concentration scales, respectively.

Analogously, for all equilibria involving water (e.g., in hydrolysis reactions), its activity must be considered in calculations. In this manuscript, the simple relationship

(10) $$a_{w}=-0.015\;I$$

was used for these purposes (valid in NaCl_(aq)_ at *T* = 298.15 K) [83].

##### Pitzer Approach

Together with SIT, the Pitzer approach is among the most widely used approaches to model the dependence of activity coefficients (and stability constants) on medium and ionic strength. For a cation “M^z+^” or an anion “X^z-^” in ionic media containing other cations “c” and anions “a”, this is

(11) $$\ln\gamma_{M}=\;z_{+}^{2}\;f^{\gamma }+2\;\sum_{a}m_{a}\left(B_{Ma}+E\;C_{Ma}\right)+\;\sum_{a}\sum_{c}m_{c}m_{a}\left(z_{+}^{2}\;B_{ca}^{\prime }+\;z_{+}^{}C_{ca}\right)+\;\;\sum_{c}m_{c}\left(2\;\Theta_{Mc}+\;\sum_{a}m_{a}\Psi_{Mca}\right)+\;\sum_{a}\sum_{a^{\prime }}m_{a}m_{a^{\prime }}\Psi_{Maa^{\prime }},$$

(12) $$\ln\gamma_{X}=\;z_{-}^{2}\;f^{\gamma }+2\;\underset{c}{\sum }m_{c}\left(B_{Xc}+E\;C_{Xc}\right)+\;\underset{a}{\sum }\underset{c}{\sum }m_{c}m_{a}\left(z_{-}^{2}\;B_{ca}^{\prime }+\;z_{-}^{}C_{ca}\right),\;+\;\underset{a}{\sum }m_{a}\left(2\;\Theta_{Xa}+\;\underset{c}{\sum }m_{c}\Psi_{Xac}\right)+\;\underset{c}{\sum }\underset{c^{\prime }}{\sum }m_{c}m_{ac^{\prime }}\Psi_{Xcc^{\prime }}$$

with

(13) $$E\;=\;1/2\sum_{i}m_{i}\left|z_{i}\right|\;,$$

(14) $$f^{\gamma }=\;-A_{\Phi }\left[\frac{\sqrt{I}}{1+1.2\;\sqrt{I}}+\frac{2}{1.2}\ln\left(1+1.2\sqrt{I}\right)\right]\;,$$

(15) $$B_{MX}=\beta_{MX}^{\left(0\right)}+\beta_{MX}^{\left(1\right)}\;f\left(\alpha_{1}\sqrt{I}\right)+\beta_{MX}^{\left(2\right)}\;f\left(\alpha_{2}\sqrt{I}\right),$$

(16) $${B^{\prime }}_{MX}=\;\frac{\beta_{MX}^{\left(1\right)}\;f^{\prime }\left(\alpha_{1}\sqrt{I}\right)+\beta_{MX}^{\left(2\right)}\;f^{\prime }\left(\alpha_{2}\sqrt{I}\right)}{I}\;,$$

(17) $$C_{MX}=\;\frac{C_{MX}^{\Phi }}{2\;\sqrt{\left|z_{M}\;z_{X}\right|}},$$

(18) $$f\left(x\right)=\;\frac{2\left[1-\left(1+x\right)\;exp\left(-x\right)\right]}{x^{2}},$$

(19) $$f^{\prime \left(x\right)}=\;\frac{-2\left[1-\left(1+x+\frac{x^{2}}{2}\right)\;exp\left(-x\right)\right]}{x^{2}},$$

where *A*_Φ_ = 0.392 and 0.399 at *T* = 298.15 and 310.15 K, respectively; α_1_ and α_2_ can vary, though very often they are α_1_ = 2.0 and α_2_ = 0.0 ((kg mol ^–1^)^1/2^) for all electrolytes except 2:2 (in which α_1_ = 1.4 and α_2_ = 12); and *β*^(0)^, *β*^(1)^, *β*^(2)^, *C*^Φ^ Θ, and Ψ are the so-called Pitzer interaction coefficients. Finally, in the Pitzer model, the activity coefficient of a neutral species in a given medium is accounted for by

(20) logγ=2 λ I.

In order to bypass difficulties related to a quite complex mathematical formulation (or when some interaction coefficients are not available), some simplified forms of Pitzer equations can be used to model the dependence of stability constants on ionic strength:

(21) $$\log\;\beta_{pqr}^{}=\log\;\beta_{pqr}^{0}+\frac{z^{*}f^{\gamma }+2p_{1}I+p_{2}I^{2}+p_{3}\left[2I\;f\left(2\sqrt{I}\right)\right]+\frac{1}{2}z^{*}\left[2I\;f\prime \left(2\sqrt{I}\right)\right]\beta_{MX}^{\left(1\right)}}{\ln10},$$

where *p*_1_ and *p*_2_ represent, for all species involved in the formation equilibrium, the summation sign of all the classical Pitzer coefficients dependent on *I* (i.e., *β*^(0)^_,_ Θ, *λ*) and *I*^2^ (i.e., *C*^Φ^, Ψ), respectively, while *p*_3_ is given by all *β*^(1)^ values.

## 4. Conclusions

The interaction of epinephrine with CH_3_Hg^+^, (CH_3_)_2_Sn^2+^, and UO_2_^2+^ was investigated in different experimental conditions in NaCl_aq_ solutions, and the main results can be summarized as follows:

- The speciation models were characterized by simple mononuclear species, except for UO_2_^2+^, where we observed the formation of the binuclear species M_2_L_2_ and M_2_L_2_(OH)_2_, which have already been obtained in several other UO_2_^2+^/oxygen donor ligand systems;
- The investigations, carried out in NaCl_aq_, allowed us to obtain the ternary (CH_3_Hg)*Eph*Cl^0^ species for the CH_3_Hg^+^/*Eph*^−^ system;
- The CH_3_Hg^+^/*Eph*^−^ species had the lowest stability compared to the (CH_3_)_2_Sn^2+^/*Eph*^−^ and UO_2_^2+^/*Eph*^−^ systems;
- For the (CH_3_)_2_Sn^2+^/*Eph*^−^ system, complexation started at acidic pH values (about 4);
- The stability and the formation percentage of these species were higher than those of the CH_3_Hg^+^/*Eph*^−^ system;
- The speciation model was formed by simple mononuclear species (MLH, ML, and MLOH), as was confirmed by UV spectrophotometry;
- Despite a fairly high stability of the complexes, the hydrolytic species of dimethyltin(IV) played a fundamental role in the speciation of the system;
- For the UO_2_^2+^/*Eph*^−^ system, the formation of binuclear complexes was observed together with the mononuclear ones;
- Complexation by epinephrine strongly reduced UO_2_^2+^ hydrolysis along the entire investigated pH range;
- By means of isoperibol calorimetry, enthalpy and entropy changes for both the protonation of epinephrine and its complexes with UO_2_^2+^ were determined;
- The obtained results highlighted that the process of formation of the UO_2_^2+^/*Eph*^−^ species was exothermic in nature and that the entropic contribution was the driving force of the reactions;
- The dependence of the stability constants on ionic strength was investigated using EDH, SIT, and Pitzer models;
- The effective sequestering ability of adrenaline toward the investigated cations was quantified by means of the pL_0.5_ parameter;
- Ionic strength had a lower effect on the sequestering ability than did pH;
- For the CH_3_Hg^+^/*Eph*^−^ system, pL_0.5_ increased up to *I* = 0.5 mol dm^−3^, and then it started to decrease from this value up to *I* = 1.0 mol dm^−3^ (as a result of chloride interactions with methylmercury).

## Figures and Tables

**Table 1:** Experimental stability constants of CH_3_Hg^+^/*Eph*^−^ complexes in NaCl_aq_ at different ionic strengths and at *T* = 298.15 K.

*I*/mol dm^−3^	log *β*_110_^1, 3^	log *β*_111_^1, 3^	log *β*_11-1_^1, 3^	log *β*_MLCl_^2, 3^
0.151	8.56 ± 0.03	17.33 ± 0.04	−0.79 ± 0.04	9.17 ± 0.07
0.502	8.38 ± 0.06	17.24 ± 0.03	−1.08 ± 0.03	8.70 ± 0.04
0.753	8.20 ± 0.04	17.14 ± 0.03	−1.27 ± 0.02	8.49 ± 0.05
0.998	8.15 ± 0.06	16.99 ± 0.04	−1.44 ± 0.04	8.44 ± 0.07

^1^ log *β*_pqr_ refers to equilibrium: p M^m+^ + q L^z−^ + r H^+^ = M_p_L_q_H_r_^(mp-zq+r)^; ^2^ log *β*_MLCl_ refers to equilibrium: M^+^ + L^−^ + Cl^−^ = MLCl^−^; ^3^ ±95% confidence interval.

**Table 2:** Experimental stability constants of (CH_3_)_2_Sn^2+^/*Eph*^−^ complexes in NaCl_aq_ at different ionic strengths and *T* = 310.15 K.

*I*/mol dm^−3^	log *β*_110_^1^	log *β*_111_^1^	log *β*_11-1_^1^
0.099	15.79 ± 0.02	20.46 ± 0.02	7.95 ± 0.01
0.149	15.60 ± 0.02	20.28 ± 0.02	7.81 ± 0.01
0.247	15.30 ± 0.01	19.98 ± 0.02	7.57 ± 0.01
0.492	14.73 ± 0.01	19.43 ± 0.02	7.13 ± 0.01
0.734	14.26 ± 0.02	18.98 ± 0.02	6.77 ± 0.01
0.974	13.83 ± 0.03	18.56 ± 0.03	6.43 ± 0.02

^1^ log *β*_pqr_ refers to equilibrium: p M^m+^ + q L^z−^ + r H^+^ = M_p_L_q_H_r_^(mp-zq+r)^; ±95% confidence interval.

**Table 3:** Experimental stability constants of UO_2_^2+^/*Eph*^−^ complexes in NaCl_aq_ at different ionic strengths and *T* = 298.15 K.

*I*/mol dm^−3^	log *β*_110_^1^	log *β*_11-1_^1^	log *β*_220_^1^	log *β*_22-2_^1^
0.146	12.35 ± 0.02	6.75 ± 0.01	27.46 ± 0.05	16.46 ± 0.02
0.491	12.16 ± 0.01	6.57 ± 0.01	26.97 ± 0.02	16.13 ± 0.01
0.736	12.03 ± 0.01	6.44 ± 0.02	26.99 ± 0.02	16.12 ± 0.01
0.982	11.96 ± 0.01	6.46 ± 0.03	26.96 ± 0.03	16.10 ± 0.01

^1^ log *β*_pqr_ refers to equilibrium: p M^m+^ + q L^z−^ + r H^+^ = M_p_L_q_H_r_^(mp-zq+r)^; ±95% confidence interval.

**Table 4:** Δ*H*, Δ*G*, and *T*Δ*S* values for *Eph*^−^ protonation and its complexes with UO_2_^2+^ in NaCl_aq_ at *I* = 0.5 mol dm^−3^ and *T* = 298.15 K.

Species ^1^	Δ*H* ^2^	Δ*G* ^2^	*T*Δ*S*^2^
LH	−39 ± 1	−57.69 ± 0.05	19 ± 3
LH_2_	−67 ± 1	−107.39 ± 0.05	40 ± 3
ML	−26 ± 4	−69.16 ± 0.02	43 ± 10
M_2_L_2_(OH)_2_	39 ± 2	−92.21 ± 0.02	131 ± 6

^1^ log *β*_pqr_ refers to equilibrium: p M^m+^ + q L^z−^ + r H^+^ = M_p_L_q_H_r_^(mp-zq+r)^; ^2^ in kJ mol^−1^; ±95% confidence interval.

**Table 5:** Stability constants of M^n+^/*Eph^−^* at infinite dilution and corresponding parameters for their dependence on ionic strength in NaCl_aq_ using the extended Debye–Hückel (EDH) and specific ion interaction theory (SIT) models at *T* = 298.15 K.

	**CH_3_Hg^+^**			
	**log *β*_110_^1, 3^**	**log *β*_111_^1, 3^**	**log *β*_11-1_^1, 3^**	**log *β*_MLCl_^2, 3^**
$log\;\beta_{pqr}^{0}$	8.98 ± 0.07	17.73 ± 0.05	−0.62 ± 0.05	9.47 ± 0.08
***C*^4^**	−0.49 ± 0.11	−0.32 ± 0.07	−0.86 ± 0.08	−0.71 ± 0.12
**Δ** ***ε*^5^**	−0.48 ± 0.08	−0.34 ± 0.04	−0.85 ± 0.06	−0.71 ± 0.10
	**UO_2_^2+^**			
	**log *β*_110_^1, 3^**	**log *β*_11-1_^1, 3^**	**log *β*_220_^1, 3^**	**log *β*_22-2_^1, 3^**
$log\;\beta_{pqr}^{0}$	12.86 ± 0.03	7.24 ± 0.01	28.16 ± 0.06	17.40 ± 0.03
***C*^4^**	−0.09 ± 0.03	0.02 ± 0.03	−0.02 ± 0.08	0.33 ± 0.03
**Δ** ***ε*^5^**	−0.09 ± 0.02	0.03 ± 0.04	−0.04 ± 0.04	0.32 ± 0.02
	**(CH_3_)_2_Sn^2+ 6^**			
	**log *β*_110_^1, 3^**	**log *β*_111_^1, 3^**	**log *β*_11-1_^1, 3^**	
$log\;\beta_{pqr}^{0}$	16.37 ± 0.04	20.81 ± 0.06	8.50 ± 0.04	
***C*^4^**	−1.79 ± 0.08	−1.95 ± 0.08	−1.30 ± 0.06	
**Δ** ***ε*^5^**	−1.75 ± 0.08	−1.91 ± 0.10	−1.26 ± 0.06	

^1^ log *β*_pqr_ refers to equilibrium: p M^m+^ + q L^z−^ + r H^+^ = M_p_L_q_H_r_^(mp-zq+r)^; ^2^ log *β*_MLCl_ refers to equilibrium: M^+^ + L^−^ + Cl^−^ = MLCl^−^; ^3^ ±95% confidence interval; ^4^ EDH equation (Equation (4)) in mol dm^−3^; ^5^ SIT equations (Equations (4) and (7)) in mol kg^−1^; ^6^ at *T* = 310.15 K.

**Table 6:** Classical SIT interaction coefficients for UO_2_^2+^/*Eph^−^* species at *T* = 298.15 K.

M^m+^	X^z−^	Neutral Species	*ε*_MX_	*k_m_* ^5^
H^+^	Cl^−^	--	0.12 ^1^	--
Na^+^	*Eph* ^−^	--	−0.219 ^2^	--
UO_2_^2+^	Cl*^−^*	--	0.25 ^3^	--
UO_2_*Eph*^+^	Cl*^−^*	--	0.12 ± 0.01 ^4^	--
--	--	UO_2_*Eph*OH	--	0.11 ± 0.02 ^4^
(UO_2_)_2_(*Eph*)_2_^2+^	Cl*^−^*	--	0.11 ± 0.02 ^4^	--
--	--	(UO_2_)_2_(*Eph*)_2_(OH)_2_	--	−0.05 ± 0.01 ^4^

^1^ Reference [61]; ^2^ Reference [17]; ^3^ Reference [55]; ^4^ ±95% confidence interval; ^5^ Setschenow coefficient of neutral species [62,63].

**Table 7:** Simplified Pitzer coefficients for the dependence on ionic strength of M^n+^/*Eph^−^* species in NaCl_aq_ at *T* = 298.15 K.

	**H^+^**			
	**log *K*_011_^1, 2^**	**log *K*_012_^1, 2^**		
$log\;K_{01r}^{0}$	10.41 ± 0.04	8.65 ± 0.02		
***p*_1_**	0.21 ± 0.03	0.93 ± 0.10		
***p*_3_**	0.11 ± 0.02	0.35 ± 0.11		
	**CH_3_Hg^+^**			
	**log *β*_110_^2, 3^**	**log *β*_111_^2, 3^**	**log *β*_11-1_^2, 3^**	**log *β*_MLCl_^2, 4^**
$log\;\beta_{pqr}^{0}$	8.98 ± 0.06	17.74 ± 0.06	−0.62 ± 0.04	9.47 ± 0.09
***p*_1_**	0.17 ± 0.10	−0.22 ± 0.13	−0.60 ± 0.08	−0.25 ± 0.12
***p*_3_**	−1.31 ± 0.24	0.37 ± 0.18	−1.15 ± 0.26	−0.86 ± 0.32
	**UO_2_^2+^**			
	**log *β*_110_^2, 3^**	**log *β*_11-1_^2, 3^**	**log *β*_220_^2, 3^**	**log *β*_22-2_^2, 3^**
$log\;\beta_{pqr}^{0}$	12.86 ± 0.04	7.24 ± 0.02	28.16 ± 0.04	17.40 ± 0.04
***p*_1_**	−0.02 ± 0.12	0.15 ± 0.10	0.58 ± 0.18	0.72 ± 0.12
***p*_3_**	1.52 ± 0.21	1.38 ± 0.30	0.70 ± 0.25	2.41 ± 0.31
	**(CH_3_)_2_Sn^2+ 5^**			
	**log *β*_110_^2, 3^**	**log *β*_111_^2, 3^**	**log *β*_11-1_^2, 3^**	
$log\;\beta_{pqr}^{0}$	16.36 ± 0.05	20.80 ± 0.07	8.50 ± 0.06	
***p*_1_**	−1.64 ± 0.13	−1.78 ± 0.16	−1.20 ± 0.09	
***p*_3_**	0.64 ± 0.23	−0.36 ± 0.18	1.01 ± 0.15	

^1^ log *K*_01r_ refers to equilibrium: H^+^ + H_r−1_L^(z-r−1)-^ = H_r_L^(z-r)-^; ^2^ ±95% confidence interval; ^3^ log *β*_pqr_ refers to equilibrium: p M^m+^ + q L^z−^ + r H^+^ = M_p_L_q_H_r_^(mp-zq+r)^; ^4^ log *β*_MLCl_ refers to equilibrium: M^+^ + L^−^ + Cl^−^ = MLCl^−^; ^5^ at *T* = 310.15 K.

**Table 8:** Classical Pitzer interaction coefficients for H^+^/*Eph^−^* and UO_2_^2+^/*Eph^−^* species at *T* = 298.15 K.

M^m+^	X^z−^	Neutral Species	*β* ^(0)^	*β* ^(1)^	*C* ^(^^φ)^	*k_m_* ^4^
H^+ 1^	Cl^−^	--	0.1775 ^2^	0.2945 ^2^	0.00080 ^2^	--
Na^+^	Cl^−^	--	0.0765 ^2^	0.2664 ^2^	0.00127 ^2^	--
Na^+^	*Eph* ^−^	--	−0.1252	0.6187	--	--
--	*--*	H*Eph*	--	--	--	−0.108
H_2_*Eph^+^*	Cl*^−^*	--	−0.0264	−0.0263	--	--
UO_2_^2+^	Cl*^−^*	--	0.4274 ^3^	1.644 ^3^	−0.03686 ^3^	--
UO_2_*Eph*^+^	Cl*^−^*	--	0.3241	0.7930	--	--
--	--	UO_2_*Eph*OH	--	--	--	0.11
(UO_2_)_2_(*Eph*)_2_^2+^	Cl*^−^*	--	0.0139	3.933	--	--
--	--	(UO_2_)_2_(*Eph*)_2_(OH)_2_	--	--	--	−0.05

^1^ θ_HNa_ = 0.036 and ψ_HNaCl_ = −0.004 in the calculations, from Reference [45]; ^2^ Reference [45]; ^3^ Reference [51]; ^4^ Setschenow coefficient of neutral species [62].

**Table 9:** Here, pL_0.5_ values for the M^n+^/*Eph*^−^ systems calculated in different experimental conditions.

*I*/mol dm^−3^	pH	pL_0.5_
**UO_2_^2+ 1^**		
0.15	5.5	4.35
0.15	7.4	7.68
0.15	8.2	8.89
0.5	7.4	7.79
0.75	7.4	7.85
1	7.4	7.89
**CH_3_Hg^+ 1^**		
0.15	9.5	2.40
0.5	9.5	2.95
0.75	9.5	2.77
1	9.5	2.64
**(CH_3_)_2_Sn^2+ 2^**		
0.15	4.0	4.76
0.15	7.4	5.64
0.15	8.2	5.97
0.15	10.0	5.99
0.50	7.4	5.15
1.00	7.4	4.59

^1^ at *T* = 298.15 K; ^2^ at *T* = 310.15 K.

**Table 10:** Literature data for some M^n+^/*Eph*^−^ systems.

M^m+^	*I* ^1^	Medium	*T/K*	Species ^2^					Ref.
				log β_110_	log β _111_	log β_121_	log β_122_	log β_120_	
Ni^2+^	0.5	NaNO_3_	293.15	--	17.43	22.9	32.0	12.5	[14]
	0.1	KCl	298.15	10.40	--	--	--	--	[65]
	0.2	KCl	298.15	9.43	18.87	24.7	34.5	14.2	[15] ^3^
Cu^2+^	0.5	NaNO_3_	293.15	--	22.64	--	43.1	23.9	[14]
	0.1	KCl	298.15	14.95	--	--	--	27.45	[66]
	0.2	KCl	298.15	15.6	23.57	34.2	44.0	24.0	[15]
Zn^2+^	0.5	NaNO_3_	293.15	--	18.26	--	--	--	[14]
	0.1	KCl	298.15	10.92	--	--	--	20.12	[65]
	0.2	KCl	298.15	--	19.75	28.35	37.99	18.19	[15]
Cd^2+^	0.5	NaNO_3_	293.15	--	16.45	--	--	--	[14]
Pb^2+^	0.5	NaNO_3_	293.15	--	21.06	--	--	--	[14]
Mn^2+^	0.1	KCl	298.15	8.80	--	--	--	15.1	[65]
	0.2	KCl	298.15	7.69	17.56	22.5	--	12.46	[15]
Co^2+^	0.1	KCl	298.15	9.61	--	--	--	16.71	[65]
	0.2	KCl	298.15	9.23	18.60	25.25	35.07	15.15	[15]
La^3+^	0.2	KCl	298.15	--	5.96	--	10.5	--	[67]
Y^3+^	0.2	KCl	298.15	--	7.40	--	13.78	--	[54]
Pr^3+^	0.15	NaCl	310.15	9.76	--	--	--	18.21	[68]
Hg^2+^	0.1	NaNO_3_	298.15	8.20	--	--	--	15.36	[65]

^1^ in mol dm^−3^; ^2^ log *β*_pqr_ refers to equilibrium: p M^m+^ + q L^z-^ + r H^+^ = M_p_L_q_H_r_^(mp-zq+r)^; ^3^ for Ni^2+^/Eph^−^ systems, the ML_2_OH species was also proposed: log β = 6.74.

## Supplementary Materials

The following are available online at https://www.mdpi.com/1420-3049/25/3/511/s1, Table S1: Hydrolysis constants of (CH_3_)_2_Sn^2+^ in NaCl_aq_ at different ionic strengths and *T* = 310.15 K; Table S2. Hydrolysis constants of UO_2_^2+^ in NaCl_aq_ at different ionic strengths and *T* = 298.15 K; Table S3: Hydrolysis constants and chloride complex ^1^ of CH_3_Hg^+^ in NaCl_aq_ at different ionic strengths and *T* = 298.15 K; Table S4: Protonation constants of epinephrine at infinite dilution and parameters for their dependence on ionic strength in NaClaq using EDH and SIT models at *T* = 298.15 and 310.15 K; Table S5: Example of experimental conditions adopted for the (CH_3_)_2_Sn^2+^/*Eph*^−^ system in NaCl_aq_ at *I* = 0.15 mol dm^−3^ and at *T* = 310.15 K; Table S6: Stability constants of UO_2_^2+^/*Ac*^−^ species in NaCl_aq_ at different ionic strengths and *T* = 298.15 K; Table S7: Stability constants of CH_3_Hg^+^/*Eph*^−^ complexes in NaCl_aq_ at different ionic strengths and *T* = 298.15 K, calculated using the EDH and SIT models; Table S8: Stability constants of (CH_3_)_2_Sn^2+^/*Eph*^−^ complexes in NaCl_aq_ at different ionic strengths and *T* = 310.15 K, calculated using the EDH and SIT models; Table S9: Stability constants of UO_2_^2+^/*Eph*^−^ complexes in NaCl_aq_ at different ionic strengths and *T* = 298.15 K, calculated using the EDH and SIT models; Table S10: Example of experimental conditions adopted for calorimetric titrations in NaCl_aq_ at *I* = 0.50 mol dm^−3^ and at *T* = 298.15 K; Figure S1: Molar absorptivity coefficients of adrenaline versus λ/nm (C_L_ = 0.09 mmol L^−1^) in NaCl_aq_ at *I* = 0.15 mol dm^−3^ and *T* = 310.15 K; Figure S2: Sequestering ability of *Eph*^−^ toward (CH_3_)_2_Sn^2+^ at *T* = 310.15 K and *I* = 0.15 mol dm^−3^; Figure S3: Sequestering ability of *Eph*^−^ toward UO_2_^2+^ at *I* = 0.15 mol dm^−3^ and at different pH values.

## References

[1] Szulczewski D.H., Hong W.H. (1978). Epinephrine. Analytical Profiles of Drug Substances.

[2] Schweigert N., Zehnder A.J., Eggen R.I. (2001). Chemical properties of catechols and their molecular modes of toxic action in cells, from microorganisms to mammals. Environ. Microbiol..

[3] Cahill L., Alkire M.T. (2003). Epinephrine enhancement of human memory consolidation: Interaction with arousal at encoding. Neurobiol. Learn. Mem..

[4] Flint R.W., Bunsey M.D., Riccio D.C. (2007). Epinephrine-induced enhancement of memory retrieval for inhibitory avoidance conditioning in preweanling Sprague-Dawley rats. Dev. Psychobiol..

[5] Jurado-Berbel P., Costa-Miserachs D., Torras-Garcia M., Coll-Andreu M., Portell-Cortes I. (2010). Standard object recognition memory and “what” and “where” components: Improvement by post-training epinephrine in highly habituated rats. Behav. Brain Res..

[6] Piperea-Sianu A., Sirbu I., Mati E., Piperea-Sianu D., Mircioiu C. (2015). Study of Synergic Effect between Some Metal Ions and Adrenaline on Human Blood Platelets Aggregation. Farmacia.

[7] Campuzano H.C., Wilkerson J.E., Horvath S.M. (1975). Fluorometric analysis of epinephrine and norepinephrine. Anal. Biochem..

[8] Grunert R., Wollmann H. (1982). The effect of ultraviolet and visible light on drugs of the phenylalkylamine series with a view toward their stability in plastic containers. Pharmazie.

[9] Bonhomme L., Benhamou D., Comoy E., Preaux N. (1990). Stability of epinephrine in alkalinized solutions. Ann. Emerg. Med..

[10] Grant T.A., Carroll R.G., Church W.H., Henry A., Prasad N.H., Abdel-Rahman A.A., Allison E.J. (1994). Environmental temperature variations cause degradations in epinephrine concentration and biological activity. Am. J. Emerg. Med..

[11] Nagy P.I., Takacs-Novak K. (2004). Tautomeric and conformational equilibria of biologically important (hydroxyphenyl)alkylamines in the gas phase and in aqueous solution. Phys. Chem. Chem. Phys..

[12] Adeniyi W.K., Wright A.R. (2009). Novel fluorimetric assay of trace analysis of epinephrine in human serum. Spectrochim. Acta A Mol. Biomol. Spectrosc..

[13] Antikainen P.J., Witikainen U. (1973). A comparative study on the ionization of catechol amines in aqueous solutions. Acta Chem. Scand..

[14] Grgas-Kužnar B., Simeon V., Weber O.A. (1974). Complexes of adrenaline and related compounds with Ni^2+^, Cu^2+^, Zn^2+^, Cd^2+^ and Pb^2+^. J. Inorg. Nucl. Chem..

[15] Gergely A., Kiss T., Deák G., Sóvágó I. (1981). Complexes of 3,4-dihydroxyphenyl derivatives IV. Equilibrium studies on some transition metal complexes formed with adrenaline and noradrenaline. Inorg. Chim. Acta.

[16] Ibrahim O.B., Mohamed M.A., Refat M.S. (2014). Study the chemical composition and biological outcomes resulting from the interaction of the hormone adrenaline with heavy elements: Infrared, Raman, electronic, 1H NMR, XRD and SEM studies. J. Mol. Struct..

[17] Bretti C., Cigala R.M., Crea F., De Stefano C., Vianelli G. (2015). Solubility and modeling acid-base properties of adrenaline in NaCl aqueous solutions at different ionic strengths and temperatures. Eur. J. Pharm. Sci..

[18] Choppin G.R., Allard B., Freeman A.J., Keller C. (1985). Complexes of actinides with naturally occurring organic compounds. Handbook on the Physics and Chemistry of Actinides.

[19] Rashan L.J., AlAllaf T.A.K. (1997). New dimethyltin (IV) compounds and their complexes with nitrogen containing ligands of antitumour activity. Eur. J. Cancer.

[20] Carrier G., Bouchard M., Brunet R.C., Caza M. (2001). A toxicokinetic model for predicting the tissue distribution and elimination of organic and inorganic mercury following exposure to methyl mercury in animals and humans. II. Application and validation of the model in humans. Toxicol. Appl. Pharm..

[21] Ullrich S.M., Tanton T.W., Abdrashitova S.A. (2001). Mercury in the Aquatic Environment: A Review of Factors Affecting Methylation. Crit. Rev. Environ. Sci. Technol..

[22] Moulin V., Moulin C. (2001). Radionuclide speciation in the environment: A review. Radiochim. Acta.

[23] Guillaumont R., Fanghänel T., Fuger J., Grenthe I., Neck V., Palmer D.A., Rand M.H. (2003). Update on the Chemical Thermodynamics of Uranium, Neptunium, Plutonium, Americium and Technetium.

[24] Salbu B., Lind O.C., Skipperud L. (2004). Radionuclide speciation and its relevance in environmental impact assessments. J. Environ. Radioact..

[25] Crea F., Foti C., Sammartano S. (2008). Sequestering ability of polycarboxylic ligands towards dioxouranium(VI). Talanta.

[26] Cigala R.M., De Stefano C., Giacalone A., Gianguzza A., Sammartano S. (2011). Hydrolysis of Monomethyl-, Dimethyl-, and Trimethyltin(IV) Cations in Fairly Concentrated Aqueous Solutions at I = 1 mol·L^−1^(NaNO_3_) and T = 298.15 K. Evidence for the Predominance of Polynuclear Species. J. Chem. Eng. Data.

[27] Berto S., Crea F., Daniele P.G., Gianguzza A., Pettignano A., Sammartano S. (2012). Advances in investigation of dioxouranium(VI) complexes of interest for natural fluids. Coord. Chem. Rev..

[28] Cataldo S., De Stefano C., Gianguzza A., Pettignano A. (2012). Sequestration of (CH_3_)Hg^+^ by amino-polycarboxylic chelating agents. J. Mol. Liq..

[29] Cataldo S., Gianguzza A., Pettignano A., Piazzese D., Sammartano S. (2012). Complex Formation of Copper(II) and Cadmium(II) with Pectin and Polygalacturonic Acid in Aqueous Solution. An ISE-H^+^ and ISE-Me^2+^ Electrochemical Study. Int. J. Electrochem. Sci..

[30] Cataldo S., De Stefano C., Gianguzza A., Pettignano A., Sammartano S. (2013). Sequestration of alkyltin(IV) cations by complexation with amino-polycarboxylic chelating agents. J. Mol. Liq..

[31] Cataldo S., Gianguzza A., Pettignano A., Villaescusa I. (2013). Mercury(II) removal from aqueous solution by sorption onto alginate, pectate and polygalacturonate calcium gel beads. A kinetic and speciation based equilibrium study. React. Funct. Polym..

[32] Lavoie R.A., Jardine T.D., Chumchal M.M., Kidd K.A., Campbell L.M. (2013). Biomagnification of mercury in aquatic food webs: A worldwide meta-analysis. Environ. Sci. Technol..

[33] Crea F., De Stefano C., Foti C., Milea D., Sammartano S. (2014). Chelating agents for the sequestration of mercury(II) and monomethyl mercury(II). Curr. Med. Chem..

[34] Piazzese D., Cataldo S., Muratore N. (2015). Voltammetric Investigation on Uranyl Sorption by Alginate Based Material. Influence of Hydrolysis and pH Dependence. Int. J. Electrochem. Sci..

[35] Crea F., Milea D., Sammartano S. (2005). Enhancement of hydrolysis through the formation of mixed hetero-metal species: Dioxouranium(VI)-cadmium(II) mixtures. Ann. Chim. (Rome).

[36] Crea F., Milea D., Sammartano S. (2005). Enhancement of hydrolysis through the formation of mixed hetero-metal species. Talanta.

[37] Crea P., De Stefano C., Milea D., Sammartano S. (2008). Formation and stability of mixed Mg^2+^/Ca^2+^/phytate species in seawater media. Consequences on ligand speciation. Mar. Chem..

[38] De Stefano C., Lando G., Milea D., Pettignano A., Sammartano S. (2010). Formation and Stability of Cadmium(II)/Phytate Complexes by Different Electrochemical Techniques. Critical Analysis of Results. J. Solut. Chem..

[39] Crea F., De Stefano C., Milea D., Sammartano S. (2018). Phytate–molybdate(VI) interactions in NaCl(aq) at different ionic strengths: Unusual behaviour of the protonated species. New J. Chem..

[40] Brønsted J.N. (1922). Studies on solubility. IV. The principle of the specific interaction of ions. J. Am. Chem. Soc..

[41] Guggenheim E.A., Turgeon J.C. (1955). Specific interaction of ions. Trans. Faraday Soc..

[42] Pitzer K.S. (1973). Thermodynamics of Electrolytes. I. Theoretical Basis and General Equations. J. Phys. Chem..

[43] Pitzer K.S., Mayorga G. (1973). Thermodynamics of Electrolytes. II. Activity and Osmotic Coefficients for Strong Electrolytes with on or Both Ions Univalent. J. Phys. Chem..

[44] Ciavatta L. (1980). The Specific Interaction Theory in Evaluating Ionic Equilibria. Ann. Chim. (Rome).

[45] Pitzer K.S. (1991). Activity Coefficients in Electrolyte Solutions.

[46] Grenthe I., Puigdomenech I. (1997). Modelling in Aquatic Chemistry.

[47] Cigala R.M., Crea F., Lando G., Milea D., Sammartano S. (2010). Solubility and acid-base properties of concentrated phytate in self-medium and in NaCl_(aq)_ at T = 298.15 K. J. Chem..

[48] Crea F., Cucinotta D., De Stefano C., Milea D., Sammartano S., Vianelli G. (2012). Modeling solubility, acid-base properties and activity coefficients of amoxicillin, ampicillin and (+)6-aminopenicillanic acid, in NaCl_(aq)_ at different ionic strengths and temperatures. Eur. J. Pharm. Sci..

[49] De Robertis A., Foti C., Patanè G., Sammartano S. (1998). Hydrolysis of (CH_3_)Hg^+^ in Different Ionic Media: Salt Effects and Complex Formation. J. Chem. Eng. Data.

[50] De Stefano C., Foti C., Gianguzza A., Martino M., Pellerito L., Sammartano S. (1996). Hydrolysis of (CH_3_)_2_Sn^2+^ in Different Ionic Media: Salt Effects and Complex Formation. J. Chem. Eng. Data.

[51] Gianguzza A., Milea D., Millero F.J., Sammartano S. (2004). Hydrolysis and chemical speciation of dioxouranium(VI) in aqueous media simulating the major ion composition of seawater. Mar. Chem..

[52] Hoellein L., Holzgrabe U. (2012). Ficts and facts of epinephrine and norepinephrine stability in injectable solutions. Int. J. Pharm..

[53] Pettit L.D., Powell K.J. IUPAC Stability Constants Database. http://publications.iupac.org/projects/posters01/pettit01.pdf.

[54] Aydin R. (2007). Study on the interaction of Yttrium(III) with adrenaline, noradrenaline, and dopamine. J. Chem. Eng. Data.

[55] Crea F., De Robertis A., Sammartano S. (2003). Dioxouranium carboxylate complexes. Formation and stability of acetate species at different ionic strengths in NaCl_aq_. Ann. Chim. (Rome).

[56] Smith R.M., Martell A.E., Chen Y. (1991). Critical-Evaluation of Stability-Constants for Nucleotide Complexes with Protons and Metal-Ions and the Accompanying Enthalpy Changes. Pure Appl. Chem..

[57] Crea F., De Stefano C., Pettignano A., Sammartano S. (2004). Hydrolysis of Dioxouranium(VI): A Calorimetric Study in NaCl(aq) and NaClO4(aq), at 25 °C. Acta.

[58] Crea F., Stefano C.D., Gianguzza A., Piazzese D., Sammartano S. (2006). Protonation of carbonate in aqueous tetraalkylammonium salts at 25 degrees C. Talanta.

[59] Bretti C., Cigala R.M., Crea F., Lando G., Sammartano S. (2014). Thermodynamics of proton binding and weak (Cl-, Na+ and K+) species formation, and activity coefficients of 1, 2-dimethyl-3-hydroxypyridin-4-one (deferiprone). J. Chem. Thermodyn..

[60] Cardiano P., Cigala R.M., Crea F., De Stefano C., Milea D., Sammartano S. (2019). Characterization of the thermodynamic properties of some benzenepolycarboxylic acids: Acid-base properties, weak complexes, total and neutral species solubility, solubility products in NaClaq, (CH3)4NClaq and Synthetic Sea Water (SSW). Fluid Phase Equilibria.

[61] Bretti C., Foti C., Porcino N., Sammartano S. (2006). SIT parameters for 1:1 electrolytes and correlation with Pitzer coefficients. J. Solut. Chem..

[62] Setschenow J.Z. (1889). Uber Die Konstitution Der Salzlosungenauf Grund Ihres Verhaltens Zu Kohlensaure. Z. Phys. Chem..

[63] Battaglia G., Cigala R.M., Crea F., Sammartano S. (2008). Solubility and Acid-Base Properties of Ethylenediaminetetraacetic Acid in Aqueous NaCl Solution at 0 <I <6 mol kg^−1^ and T = 298.15 K. J. Chem. Eng. Data.

[64] Crea F., De Stefano C., Irto A., Milea D., Pettignano A., Sammartano S. (2017). Modeling the Acid-Base Properties of Molybdate(VI) in Different Ionic Media, Ionic Strengths and Temperatures, by EDH, SIT and Pitzer Equations. J. Mol. Liq..

[65] Jameson R.F., Neillie W.F.S. (1966). Complexes formed by adrenaline and related compounds with transition-metal ions—III. J. Inorg. Nucl. Chem..

[66] Jameson R.F., Neillie W.F.S. (1965). Complexes formed by adrenaline and related compounds with transition-metal ions—II complexes with copper(II). J. Inorg. Nucl. Chem..

[67] Aydin R., Inci D. (2012). Potentiometric and Spectrophotometric Studies of the Complexation of Lanthanum(III) with Adrenaline, Noradrenaline, and Dopamine. J. Chem. Eng. Data.

[68] Gao F., Han J.F., Wu Z.J., Wang Y., Yang K.Y., Niu C.J., Ni J.Z. (1999). Studies on the coordination of Pr(III) with adrenaline by potentiometry and absorption spectroscopy. Chin. Chem. Lett..

[69] Jameson R.F., Neillie W.F. (1965). Complexes Formed by Adrenaline and Related Compounds with Transition-Metal Ions. I. Acid Dissociation Constants of the Ligands. J. Chem. Soc..

[70] Marzotto A., Mazzucco E., Galzigna L. (1980). Spectral changes following the epinephrine-dioxouranium(VI) interaction. Inorg. Nucl. Chem. Lett..

[71] Moustafa M.H. (2010). Studies on the binary and ternary comlexes of mercury(II) with gallic acid and adrenaline. Ass. Univ. Bull. Environ. Res..

[72] De Stefano C., Foti C., Giuffrè O., Sammartano S. (2001). Dependence on ionic strength of protonation enthalpies of polycarboxylic anions in NaCl aqueous solution. J. Chem. Eng. Data.

[73] De Stefano C., Sammartano S., Mineo P., Rigano C., Gianguzza A., Pelizzetti E., Sammartano S. (1997). Computer Tools for the Speciation of Natural Fluids. Marine Chemistry—An Environmental Analytical Chemistry Approach.

[74] Gans P. Hyperquad. http://www.hyperquad.co.uk/.

[75] De Stefano C., Princi P., Rigano C., Sammartano S. (1989). The Calculation of Equilibrium Concentrations. ES4EC1: A Fortran Program for Computing Distribution Diagrams and Titration Curves. Comput. Chem..

[76] De Robertis A., De Stefano C., Rigano C. (1989). Computer Analysis of Equilibrium Data in Solution. ES5CM Fortran and Basic Programs for Computing Formation Enthalpies from Calorimetric Measurements. Acta.

[77] Crea F., De Stefano C., Giuffrè O., Sammartano S. (2004). Ionic strength dependence of protonation constants of N-Alkylsubstituted open chain diamines in NaCl_(aq)_. J. Chem. Eng. Data.

[78] Bretti C., Giacalone A., Gianguzza A., Milea D., Sammartano S. (2007). Modeling S-carboxymethyl-L-cysteine protonation and activity coefficients in sodium and tetramethylammonium chloride aqueous solutions by SIT and Pitzer equations. Fluid Phase Equilib..

[79] Crea P., De Robertis A., De Stefano C., Milea D., Sammartano S. (2007). Modelling the dependence on medium and ionic strength of glutathione acid-base behavior in LiCl_aq_, NaCl_aq_, KCl_aq_, CaCl_aq_, (CH_3_)_4_NCl_aq_ and (C_2_H_5_)_4_NI_aq_. J. Chem. Eng. Data.

[80] Cigala R.M., Cordaro M., Crea F., De Stefano C., Fracassetti V., Marchesi M., Milea D., Sammartano S. (2014). Acid–Base Properties and Alkali and Alkaline Earth Metal Complex Formation in Aqueous Solution of Diethylenetriamine-N,N,N′,N″,N″-pentakis(methylenephosphonic acid) Obtained by an Efficient Synthetic Procedure. Ind. Eng. Chem. Res..

[81] Bretti C., Cigala R.M., Lando G., Milea D., Sammartano S. (2014). Some Thermodynamic Properties of Aqueous 2-Mercaptopyridine-N-Oxide (Pyrithione) Solutions. J. Solut. Chem..

[82] Cataldo S., Crea F., Gianguzza A., Pettignano A., Piazzese D. (2009). Solubility and acid-base properties and activity coefficients of chitosan in different ionic media and at different ionic strengths, at T = 25 °C. J. Mol. Liq..

[83] Foti C., Gianguzza A., Milea D., Sammartano S. (2002). Hydrolysis and chemical speciation of (C_2_H_5_)_2_Sn^2+^, (C_2_H_5_)_3_Sn^+^ and (C_3_H_7_)_3_Sn^+^ in aqueous media simulating the major composition of natural waters. Appl. Organomet. Chem..

