# Supplementary material for: Understanding the Solution Behavior of Epinephrine in the Presence of Toxic Cations: A Thermodynamic Investigation in Different Experimental Conditions
Source: Molecules. 2020 Jan 24;25(3):511. doi: 10.3390/molecules25030511 (PMC7037007; doi:10.3390/molecules25030511)
Supplement: Supplementary file 1 [file molecules-25-00511-s001.pdf]

molecules

Table S1. Hydrolysis constants [1] of $(CH_3)_2Sn^{2+}$ in $NaCl_{aq}$ at different ionic strengths and T = 310.15 K.

I / mol dm^{-3}	log β_{10-1} [2]	log β_{10-2} [2]	log β_{10-3} [2]	log β_{20-2} [2]	log β_{20-3} [2]
0.15	-2.99	-8.13	-18.86	-4.92	-9.19
0.50	-3.27	-8.50	-19.19	-5.22	-9.83
0.75	-3.42	-8.72	-19.48	-5.36	-10.21
1.00	-3.57	-8.92	-19.72	-5.55	-10.54

[1] Ref. [50]; [2] log β_{pqr} refer to equilibrium: p M^{m+} + q L^{z-} + r H^+ = $M_pL_qH_r^{(mp-zq+r)}$.

Table S2. Hydrolysis constants [1] of UO_2^{2+} in $NaCl_{aq}$ at different ionic strengths and T = 298.15 K.

I / mol dm^{-3}	log β_{10-1} [2]	log β_{20-2} [2]	log β_{30-4} [2]	log β_{30-5} [2]	log β_{30-7} [2]
0.15	-5.50	-6.02	-12.27	-16.64	-29.72
0.50	-5.72	-6.14	-12.40	-16.95	-29.84
0.75	-5.84	-6.19	-12.41	-17.05	-29.86
1.00	-5.96	-6.24	-12.40	-17.13	-29.85

[1] Ref. [51]; [2] log β_{pqr} refer to equilibrium: p M^{m+} + q L^{z-} + r H^+ = $M_pL_qH_r^{(mp-zq+r)}$.

Table S3. Hydrolysis constants [1] and chloride complex [1] of CH_3Hg^+ in $NaCl_{aq}$ at different ionic strengths and T = 298.15 K.

I / mol dm^{-3}	log β_{10-1} [2]	log β_{MCl} [3]
0.15	-5.50	-6.02
0.50	-5.72	-6.14
0.75	-5.84	-6.19
1.00	-5.96	-6.24

[1] Ref. [49]; [2] log β_{pqr} refer to equilibrium: p M^{m+} + q L^{z-} + r H^+ = $M_pL_qH_r^{(mp-zq+r)}$; [3] log β_{MCl} refer to equilibrium: M^{m+} + Cl^- = $MCl^{(m-1)}$.

Table S4. Protonation constants of epinephrine at infinite dilution and parameters for their dependence on ionic strength in NaClaq by EDH and SIT models, at T = 298.15 and 310.15 K [1].

	log K_{011} [2]	log K_{012} [2]
	**T = 298.15 K**	
log K^0_{01r}	10.68	8.95
C [3]	0.256	0.210
$\Delta\varepsilon$ [4]	0.267	0.225
	**T = 310.15 K**	
log K^0_{01r}	10.36	8.77
C [3]	0.186	0.308
$\Delta\varepsilon$ [4]	0.194	0.317

[1] Ref. [17]; [2] log K_{01r} refer to equilibrium: H^+ + $H_{r-1}L^{(z-r-1)-}$ = $H_rL^{(z-r)-}$; [3] EDH equation (eq. 4), in mol dm^{-3}; [4] SIT equations (eq. 4, 7), in mol kg^{-1};.

Table S5. Example of experimental conditions adopted for the $(CH_3)_2Sn^{2+}$/*Eph*$^-$ system, in $NaCl_{aq}$ at I = 0.15 mol dm^{-3} and at T = 310.15 K.

Run	Vessel			Titrant	n [3]	pH [4]	m [5]
**Potentiometry**							
	**M** [1]	**L** [1]	**H** [1]	**H** [2]			
1	1	1	10	-0.1008	80	2.4-11.00	2
2	1	2	8	-0.1008	100	2.6-11.20	3
3	2	2	8	-0.0994	96	2.5-11.10	2
4	2.5	4	8	-0.0994	94	2.5-11.00	2
5	1	4	6	-0.0994	110	2.8-10.80	1
**Spectrophotometry**							
	**M**	**L**	**H**	**H**			
1	0.03	0.05	8	-0.1241	60	2.38-11.21	3
2	0.05	0.12	8	-0.1241	57	2.49-10.81	2
3	0.05	0.12	8	-0.1241	57	2.49-10.81	2
4	0.06	0.09	8	-0.1241	55	2.50-10.90	3

[1] in mmol dm^{-3}; [2] in mol dm^{-3}; [3] average number of experimental points collected; [4] investigated pH range; [5] number of runs per condition.

Table S6. Stability constants [1] of UO_2^{2+} / *Ac*$^-$ species in $NaCl_{aq}$ at different ionic strengths and T = 298.15 K [1].

I / mol dm^{-3}	log β_{110} [2]	log β_{120} [2]	log β_{130} [2]	log β_{13-1} [2]
0.15	2.44	4.02	6.58	1.92
0.50	2.38	4.09	6.44	1.79
0.75	2.36	4.02	6.43	1.77
1.00	2.33	4.24	6.40	1.78

[1] Ref. [55]; [2] log β_{pqr} refer to equilibrium: p UO_2^{2+} + q Ac^- + r H^+ = $(UO_2)_pAc_qH_r^{(2p-q+r)}$.

Table S7. Stability constants of CH_3Hg^+/ *Eph*$^-$ complexes in $NaCl_{aq}$ at different ionic strengths and T = 298.15 K, calculated by EDH and SIT models.

I	log β_{110} [1, 3]	log β_{111} [1, 3]	log β_{11-1} [1, 3]	log β_{MLCl} [2, 3]
		**mol dm^{-3}**		
0.15	8.66 ± 0.06	17.44 ± 0.04	-0.75 ± 0.04	9.12 ± 0.06
0.50	8.39 ± 0.03	17.23 ± 0.03	-1.05 ± 0.03	8.77 ± 0.04
0.75	8.24 ± 0.04	17.11 ± 0.03	-1.26 ± 0.02	8.56 ± 0.05
1.00	8.09 ± 0.06	17.01 ± 0.04	-1.48 ± 0.04	8.35 ± 0.07
		**mol kg^{-1}**		
0.15	8.66 ± 0.01	17.44 ± 0.01	-0.74 ± 0.01	9.11 ± 0.01
0.50	8.39 ± 0.02	17.22 ± 0.01	-1.04 ± 0.01	8.76 ± 0.03
0.75	8.24 ± 0.03	17.10 ± 0.02	-1.25 ± 0.02	8.55 ± 0.04
1.00	8.09 ± 0.04	17.00 ± 0.02	-1.47 ± 0.03	8.35 ± 0.05

[1] log β_{pqr} refer to equilibrium: p M^{m+} + q L^{z-} + r H^+ = $M_pL_qH_r^{(mp-zq+r)}$; [2] log β_{MLCl} refer to equilibrium: M^+ + L^- + Cl^- = $MLCl^-$; [3] ± 95% confidence interval.

Table S8. Stability constants of $(CH_3)_2Sn^{2+}$/ *Eph*⁻ complexes in $NaCl_{aq}$ at different ionic strengths and T = 310.15 K, calculated by EDH and SIT models.

I	log β_{110} [1]	log β_{111} [1]	log β_{11-1} [1]
	**mol dm⁻³**		
0.15	15.59 ± 0.02	20.26 ± 0.02	7.80 ± 0.01
0.50	14.76 ± 0.01	19.48 ± 0.02	7.14 ± 0.01
0.75	14.24 ± 0.02	18.96 ± 0.02	6.75 ± 0.01
1.00	13.74 ± 0.03	18.45 ± 0.03	6.37 ± 0.02
	**mol kg⁻¹**		
0.15	15.59 ± 0.02	20.26 ± 0.02	7.80 ± 0.01
0.50	14.77 ± 0.01	19.49 ± 0.02	7.16 ± 0.01
0.75	14.26 ± 0.02	18.98 ± 0.02	6.77 ± 0.01
1.00	13.78 ± 0.03	18.48 ± 0.03	6.41 ± 0.02

[1] log β_{pqr} refer to equilibrium: p M^{m+} + q L^{z-} + r H^+ = $M_pL_qH_r^{(mp-zq+r)}$; ± 95% confidence interval.

Table S9. Stability constants of UO_2^{2+}/ *Eph*⁻ complexes in $NaCl_{aq}$ at different ionic strengths and T = 298.15 K, calculated by EDH and SIT models.

I	log β_{110} [1]	log β_{11-1} [1]	log β_{220} [1]	log β_{22-2} [1]
		**mol dm⁻³**		
0.15	12.35 ± 0.02	6.75 ± 0.01	27.40 ± 0.05	16.45 ± 0.02
0.50	12.12 ± 0.01	6.55 ± 0.01	27.10 ± 0.02	16.16 ± 0.01
0.75	12.03 ± 0.01	6.49 ± 0.02	26.99 ± 0.02	16.11 ± 0.01
1.00	11.96 ± 0.01	6.44 ± 0.03	26.92 ± 0.03	16.10 ± 0.01
		**mol kg⁻¹**		
0.15	12.35 ± 0.01	6.74 ± 0.01	27.40 ± 0.01	16.45 ± 0.01
0.50	12.11 ± 0.01	6.55 ± 0.01	27.09 ± 0.01	16.16 ± 0.01
0.75	12.02 ± 0.01	6.49 ± 0.02	26.97 ± 0.01	16.10 ± 0.01
1.00	11.95 ± 0.01	6.45 ± 0.02	26.89 ± 0.02	16.09 ± 0.01

[1] log β_{pqr} refer to equilibrium: p M^{m+} + q L^{z-} + r H^+ = $M_pL_qH_r^{(mp-zq+r)}$; ± 95% confidence interval.

Table S10. Example of experimental conditions adopted for the calorimetric titrations in $NaCl_{aq}$ at I = 0.50 mol dm⁻³ and at T = 298.15 K.

**Run**	**Vessel**			**Titrant**	**n** [3]	**pH** [4]	**m** [5]
				**Protonation**			
	**M** [1]	**L** [1]	**H** [1]	**H** [2]			
1	-	5	-10	0.5133	20	4.8-10.50	3
2	-	4	-8	0.5133	20	4.9-10.30	3
3	-	5	-10	0.5133	20	4.8-10.50	3
				**UO_2^{2+}**			
	**M** [1]	**L** [1]	**H** [1]	**M** [2]			
1	-	5	-10	0.0699	20	5.7-10.70	3
2	-	6	-9	0.0556	20	4.5-8.60	3
3	-	4	-8	0.0699	20	5.0-9.50	3

[1] in mmol dm⁻³; [2] in mol dm⁻³; [3] average number of experimental points collected; [4] investigated pH range; [5] number of runs per condition.

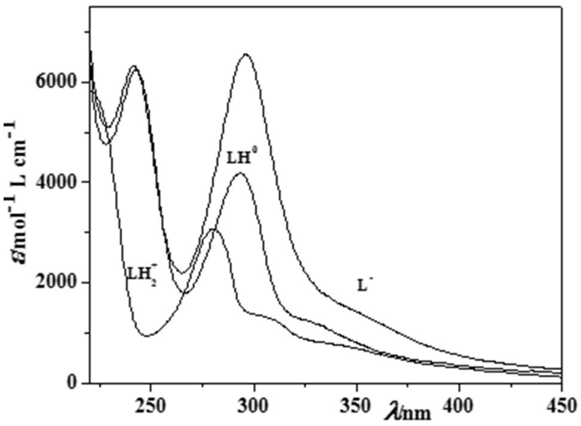

Figure S1. Molar absorptivity coefficients of adrenaline vs. λ/nm (C_L = 0.09 mmol L^{-1}) in $NaCl_{aq}$ at I = 0.15 mol dm^{-3} and T = 310.15 K.

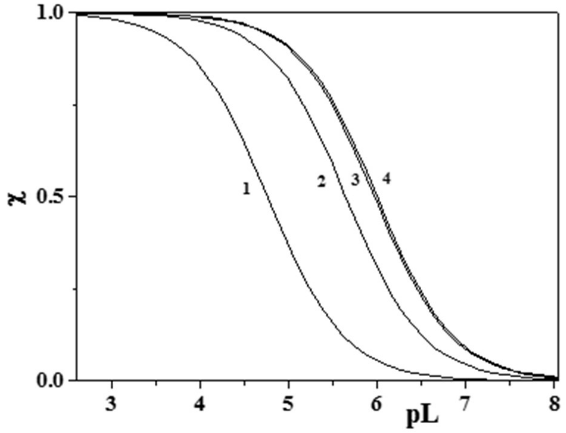

Figure S2. Sequestering ability of Eph^- towards $(CH_3)_2Sn^{2+}$ at T = 310.15 K and I = 0.15 mol dm^{-3}. Legend: 1. pH = 4.00, $pL_{0.5}$ = 4.76; 2 pH = 7.4, $pL_{0.5}$ = 5.64, 3 pH = 8.2, $pL_{0.5}$ = 5.97 and 4. pH = 10.0, $pL_{0.5}$ = 5.99.

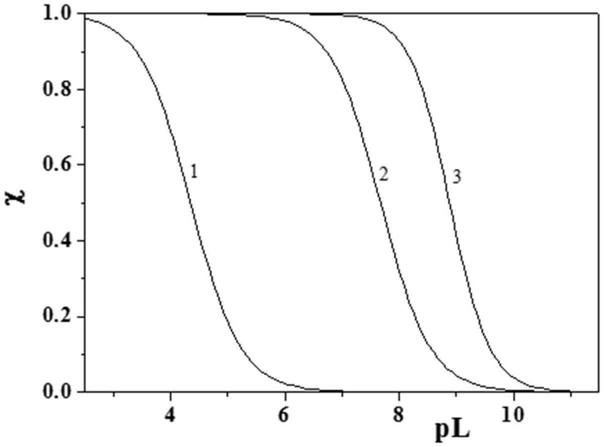

Figure S3. Sequestering ability of Eph^- towards UO_2^{2+} at I = 0.15 mol dm^{-3} and different pH values. 1. pH = 5.5, $pL_{0.5}$ = 4.35; 2. pH = 7.4, $pL_{0.5}$ = 7.68; pH = 8.2, $pL_{0.5}$ = 8.89.
